# Molecular Insight into the Effect of HIV-TAT Protein on Amyloid-β Peptides

**DOI:** 10.1021/acsomega.4c02643

**Published:** 2024-06-13

**Authors:** Asis K. Jana, Recep Keskin, Fatih Yaşar

**Affiliations:** † Department of Microbiology and Biotechnology, Sister Nivedita University, Kolkata 700156, India; ‡ Department of Physics Engineering, Hacettepe University, Ankara 06800, Türkiye

## Abstract

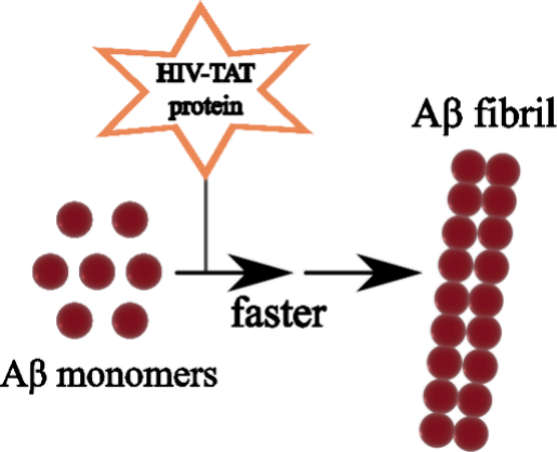

Increased deposition of amyloid-β (Aβ) plaques in the brain is a frequent pathological feature observed in human immunodeficiency virus (HIV)-positive patients. Emerging evidence indicates that HIV regulatory proteins, particularly the transactivator of transcription (TAT) protein, could interact with Aβ peptide, accelerating the formation of Aβ plaques in the brain and potentially contributing to the onset of Alzheimer’s disease in individuals with HIV infection. Nevertheless, the molecular mechanisms underlying these processes remain unclear. In the present study, we have used long all-atom molecular dynamics simulations to probe the direct interactions between the TAT protein and Aβ peptide at the molecular level. Sampling over 28.0 μs, our simulations show that TAT protein induces a shift in the Aβ monomer ensemble toward elongated conformations, exposing aggregation-prone regions on the surface and thereby inducing subsequent aggregation. TAT protein also appears to enhance the stability of preformed Aβ fibrils, while increasing the β-sheet content within these fibrils. Our atomistically detailed simulations qualitatively agree with previous in vitro and in vivo studies. Importantly, our simulations identify key interactions between Aβ and the TAT protein that drive the Aβ aggregation process and stabilize the preformed Aβ aggregates, which are particularly challenging to obtain through current experimental techniques.

## Introduction

The human immunodeficiency virus (HIV) is a retrovirus that has infected millions of people all over the world. According to the World Health Organization (WHO), as of 2022, approximately 39 million people globally are living with HIV, and around 1.3 million new infections are reported in the same year. Despite these alarming numbers, the number of new infections and related deaths is declining, mainly due to the advent of combination antiretrovirals (ARVs). The increased lifespan of HIV-positive individuals on antiretroviral therapy (ART) has led to the emergence of other comorbid medical complications,^1^ including neurocognitive dysfunction.^2^ In fact, almost 50% of HIV patients experience neurocognitive dysfunction, regardless of using combined antiretroviral therapy (cART),^2^ with a higher incidence in older individuals.^3,4^ One probable reason for neuronal injury in HIV-infected individuals could be the presence of an HIV reservoir in the brain, which is also detected in HIV-positive patients receiving antiretroviral treatment.^5,6^ Several HIV regulatory proteins such as TAT, Gp120, Vpr, and Nef, can exert direct influences on the nervous system, triggering neuroinflammatory pathways that lead to neuronal dysfunction.^7−12^ Furthermore, HIV-infected individuals exhibit an increased deposition of amyloid-β (Aβ) plaques in the brain,^13,14^ a well-known pathological feature also associated with Alzheimer’s disease (AD).^15^ Although there is no definitive evidence of a direct causal link between HIV infections and the onset of AD, growing evidence suggests that common pathways and factors are modulated in the brains of both HIV-positive and AD patients, implying potential similarities between these two pathologies.^16,17^ A key difference is that in HIV-infected individuals, Aβ plaques tend to be diffuse and commonly found inside neurons,^18−21^ unlike Alzheimer’s disease (AD), where the Aβ plaques are very dense, predominantly extracellular. Nevertheless, diffuse plaques are also detected in the early stages of AD,^22,23^ once again suggesting similarities and convergences of these two pathologies.

Mounting evidence suggests that HIV regulatory proteins, particularly the TAT protein, directly or indirectly influence the regulation of the amyloid pathway.^24−27^ TAT is a small protein comprising 86 to 101 amino acids, encoded by the TAT gene.^28,29^ It is one of the first proteins to be expressed in HIV-infected cells and functions as a key activator of HIV transcription.^30^ Notably, TAT protein can still be secreted by replicating proviral DNA, even when brain-penetrant antiretroviral drugs successfully reduce viral replication.^6^ The protein is encoded by two exons,^28^ with the first encoding the most active region of the protein (residues 1–72), which includes a proline-rich region (residues 1–21), a cysteine-rich region (residues 22–37), a hydrophobic core motif (residues 38–48), a basic domain enriched in arginine and lysine residue (residues 49–59), and a glutamine-rich domain (residues 60–72). The second exon encodes the C-terminal region (residues 73–101), containing the RGD motif essential for binding the integrin receptor.^31^ TAT accelerates the amyloid pathway through several mechanisms, including enhanced Aβ generation by disrupting endolysosome structure and function,^24,25^ as well as lessened Aβ degradation by inhibiting neprilysin (NEP), a major Aβ peptide degrading enzyme in the brain.^26,27^

Since TAT protein is abundantly released into the extracellular space,^29^ where it can interact with extracellular Aβ peptide. Hategan et al. studied the direct interaction between Aβ40 and the most active region of the TAT protein (residues 1–72), employing both theoretical and experimental approaches.^32^ They observed the Aβ40-TAT complex formation both in vitro and in animal models. Importantly, the study unveiled that TAT directly binds to the external surfaces of the Aβ fibrils, inducing increased β-sheet formation and lateral aggregation into dense multifibrillar structures and subsequently forming fibers with increased rigidity. Concurrently, Aβ40-TAT aggregates exhibit enhanced neurotoxicity, with the increase in TAT content in the fibril leading to more neuronal damage, as TAT binds more strongly to the neuronal cell membrane. A later study^33^ into the aggregation characteristics of the C-terminal fragment of Aβ (residues 33–42) in the presence of the full-length TAT protein (residues 1–101) revealed a similar finding. While these studies provide substantial insight into the effect of the TAT protein on the Aβ aggregation process, a detailed molecular understanding of this effect still remains elusive. The large conformational heterogeneity and high aggregation propensity of Aβ monomer in aqueous media pose a huge challenge in probing the mechanistic aspects of the aggregation process using current experimental techniques.^34^ In this aspect, computational and theoretical methods, particularly molecular dynamics (MD) simulations, have proven beneficial, providing valuable mechanistic insight into the self-assembly pathway of amyloidogenic peptides over the past two decades.^34−42^ Note that, our previous research group has extensively used MD simulations to study the interactions between SARS-COV-2 protein fragments and various amyloid-forming proteins.^43−46^ In the present study, we employ long unbiased atomistic MD simulations to examine the effect of the TAT protein on the Aβ monomer as well as preformed Aβ fibril, i.e., the initial and final states of the Aβ aggregation pathway, aiming to gain direct mechanistic insight. Our simulations identify key interactions between Aβ and the TAT protein which drives the Aβ aggregation process and stabilizes the preformed Aβ aggregates.

## Materials and Methods

### System Preparation

**Figure 1:**
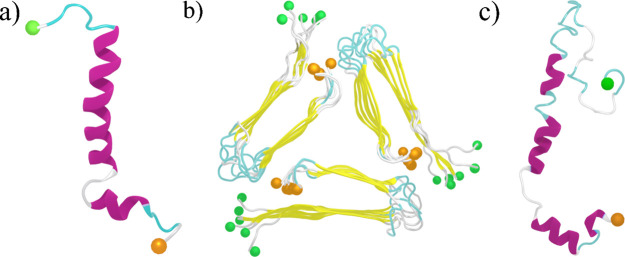
Initial configuration of (a) Aβ40 monomer, (b) Aβ40 fibril, and (c) TAT protein used in this study. Helix, β-sheet, turn and coil regions are colored in purple, yellow, cyan and white, respectively. The N- and C-terminal residues are indicated by green and orange spheres, respectively.

To understand the effect of the TAT protein on Aβ aggregation, we performed MD simulations on the Aβ40 monomer and fibril in the presence/absence of the TAT protein. Note that, we have chosen Aβ40, as it is the most abundant isoform of Aβ peptide detected in diseased cerebrospinal fluid and plasma,^47^ and also to compare our simulation results with a previous experimental study on Aβ40.^32^ The initial structure of Aβ40 monomer was retrieved from the PDB database (PDB ID: 1Z0Q),^48^ reported via solution NMR study in the “lipid-mimicking” environment of a 3:7 mixture of water and hexafluoro-2-propanol. The structure comprises 42 amino acid residues with two helical regions: a long N-terminal helix (S_8_GYEVHHQKLVFFAEDVG_25_) and a shorter, C-terminal helix (K_28_GAIIGLMVGG_38_), connected by a two-residue turn (S_26_N_27_). Notably, this structure has been employed in a number of studies to understand the conformational dynamics of Aβ monomer in water and in the interfacial environment. To generate the Aβ40 monomer structure, we deleted Ile41 and Ala42 residues from the C-terminal end. The initial coordinate of the Aβ40 fibril was taken from the solid-state NMR structure (PDB ID: 2LMQ),^49^ which exhibits 3-fold symmetry and is composed of three cross-β units. Each cross-β subunit included six identical peptides, with each peptide consisting of two β-strands: β1 (residues Y11–E22) at the N-terminus and β2 (residues A30-V39) at the C-terminus, connected by a U-bent turn. The disordered N-terminal residues (residues D1-S8) were missing in this structure. For the TAT protein, we have considered the most active region of the TAT protein (residues 1–72) and generated the initial configuration via the I-TASSER server,^50^ with the crystal structure of TAT protein (PDB ID: 3MI9)^51^ used as template. This structure was cocrystallized in a complex with a positive transcription elongation factor (P-TEFb) under milder conditions, with the first 49 residues resolved within it. We did not choose other structures of TAT protein available in the protein database, as those are derived at low pH and in extremely highly reducing environments.^52−54^ The initial configuration of Aβ40 monomer and fibril and TAT protein used in this study are depicted in Figure 1. Using the ClusPro server,^55^ we have generated an initial configuration for simulations involving TAT protein, by docking it with Aβ monomer and fibril, at ratios of 1:1 and 1:6, respectively. Simulations starting only from the Aβ monomer/fibril (i.e., without TAT protein) served as a control for comparisons against simulations where the TAT protein was present. In all systems, the N- and C-termini of Aβ peptide and TAT protein were capped by NH3^+^ and COO^–^ groups, respectively. Prior to the simulation, each system was embedded in a periodic water box, such that the minimum distance between any protein atom and a box edge was at least 15 Å. Each system was neutralized by the addition of Na^+^/Cl^–^ ions, and additional Na^+^ and Cl^–^ ions were added to attain a physiological ion concentration of 150 mM NaCl. Details of the simulation setup for all systems are given in Table 1.

**Table 1:** Simulation Details

system description		total number of atoms	number of water molecules	trials	simulation length	total sampling
Aβ monomer	control	56,331	18,542	3	4.0 μs	12.0 μs
	with TAT	84,133	27,399	3	5.0 μs	15.0 μs
Aβ fibril	control	114,792	35,326	3	0.2 μs	0.6 μs
	with TAT	235,462	74,303	3	0.2 μs	0.6 μs

### Simulation Methods

MD simulations were carried out with the GROMACS 2022.2 simulation package^56^ employing the CHARMM 36m all-atom force field^57^ and TIP3P water model.^58^ Each system was first subjected to energy minimization using the steepest descent algorithm for 50,000 steps to remove any unnatural clashes. Following this, the systems were equilibrated under NVT (310 K) conditions for 100 ps, followed by 100 ps of NPT (310 K, 1 atm). In the NVT and NPT equilibrations, the nonhydrogen (heavy) atoms of the protein were positionally restrained with a force constant of 1000 kJ mol^–1^ nm^–2^, allowing water molecules to equilibrate around the solute. Production simulations were then performed in the NPT (310 K, 1 atm) ensemble with a simulation time step of 2 fs. The temperature was maintained at 310 K by the v-rescale thermostat^59^ with a coupling constant of 0.1 ps, and the pressure was kept constant at 1 atm using the Parrinello–Rahman barostat^60^ with a coupling constant of 5 ps. The SHAKE algorithm^61^ was employed to enforce rigidity in water molecules, while the LINCS algorithm^62^ was used to constrain nonwater covalent bonds involving hydrogen atoms. Long-range electrostatic interactions were computed using the smooth particle-mesh Ewald (PME)^63^ technique with a 12 Å cutoff, and the cutoff distance for short-range Lennard-Jones (LJ) interactions was set to 12 Å, smoothened at a distance of 10.5 Å.

### Analysis Tools and Protocols

We analyzed the simulation trajectories using GROMACS tools, visual molecular dynamics (VMD),^64^ and MDTraj,^65^ and visualized the trajectories using VMD^64^ and PyMOL software.^66^ MDTraj was used to compute residue-wise chemical shifts. The radius of gyration was calculated using the GROMACS utility gmx gyrate, and secondary structure analysis was performed using the Dictionary of Secondary Structure in Proteins^67^ implemented in the GROMACS do_dssp tool. We computed the root-mean-square deviation (RMSD) and root-mean-square fluctuation (RMSF) for all backbone atoms in the Aβ fibril, relative to the experimentally solved NMR structure, using the GROMACS tools gmx rms and gmx rmsf, respectively. Before the RMSD and RMSF calculations, the rotational and translational superposition of the Aβ fibril with respect to its initial structure is performed. Residue–residue contact maps were generated using VMD, defining contacts by a 7.0 Å cutoff in the closest distance between heavy atoms within a residue pair. The total number of interpeptide hydrogen bonds was also estimated with VMD, with hydrogen bonds defined by a distance cutoff of 3.0 Å between donor and acceptor atoms and an angle between the donor-hydrogen···acceptor atoms greater than 160°. The binding effective energies (Δ*G*_eff_) of the TAT protein with Aβ and per-residue contribution to Δ*G*_eff_ were obtained using the molecular mechanics/generalized Born (Poisson–Boltzmann) surface area (MM/GB(PB)SA) method, as implemented in the gmx_MMPBSA package,^68^ which utilizes the MMPBSA.py module^69^ in AmberTools.^70^ The total free energy of the system is,
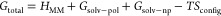


The term *H*_MM_ is defined as
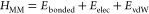


Here, *E*_bonded_ represents the bond, angle, and torsional angle energies, and *E*_elec_ and *E*_vdW_ are the electrostatic and van der Waals energies, respectively. The polar solvation free energy, *G*_solv-pol_, is calculated using the generalized Born (GB) implicit solvent model^71^ at the solvent dielectric constant of water at 310 K, i.e., ε = 78.^72^ The nonpolar solvation free energy, *G*_solv-np_, is estimated according to
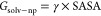
where γ and SASA are the surface tension of water (γ = 0.0072) and solvent-accessible surface area of the solute, respectively. The final term, *TS*_config_, represents the protein configurational entropy. The binding free energy (Δ*G*_binding_) of the TAT protein with Aβ is estimated as the difference:
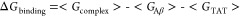


where *G*_complex_ represents the total free energy of the Aβ-TAT protein complex, and *G*_Aβ_ and *G*_TAT_ represent the total free energies of the isolated Aβ and TAT protein in the solvent, respectively. In the MM-GBSA method, entropy is usually estimated through computationally intensive normal-mode analysis, which introduces significant statistical uncertainty into the result.^73,74^ Another approach, the quasi-harmonic approximation, is computationally less expensive but often faces challenges to reach convergence.^75^ Hence, similar to many computational studies,^76−78^ we also omitted the entropic contribution in the binding free energy calculation; this then results in binding effective energy (Δ*G*_eff_ = gas-phase energy + solvation free energy). Moreover, as we have employed a single trajectory approach, whereas both the TAT protein and Aβ are extracted directly from the Aβ simulations involving the TAT protein, thus the contribution of *E*_bonded_ to Δ*G*_eff_ is identically zero. Hence, the binding effective energy is computed according to
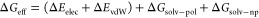


To estimate the per-residue contribution to binding effective energy, Δ*G*_eff_ is further decomposed according to the standard scheme:
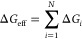


Here, *N* is the total number of residues, while Δ*G*_*i*_ is the per-residue contribution to Δ*G*_eff_.

## Results and Discussion

### Validation of Simulations with Experiment

Prior to examining the effect of the TAT protein on Aβ, we first validated the conformational ensemble obtained from our simulations with experiments. For this purpose, we have computed NMR chemical shifts (δ_sim_) for the backbone and C_β_ atoms of Aβ using SHIFTX2 program^79^ in MDTraj,^65^ and then compared with the experimentally determined NMR chemical shifts (δ_exp_). The residue-wise correlation plots are presented in Figure S1 in the Supporting Information. As depicted, the calculated C_α_, N, and C_β_ chemical shifts of the Aβ40 monomer, derived from the MD ensemble, exhibit a high degree of correlation with experimental NMR chemical shifts of the Aβ40 monomer in 100% water environment,^80^ with Pearson correlation coefficients (*R*) of 0.99, 0.97 and 0.99, respectively. Importantly, similar correlation values have been reported in numerous previous simulation studies of the Aβ40 monomer.^81−83^ Furthermore, we have computed root-mean-square deviation (RMSD) between calculated and experimentally determined NMR chemical shifts. This metric quantifies how closely chemical shift values derived from our simulations agree with experimentally measured data and has been used in numerous biomolecular simulation studies to validate simulated ensembles.^44,84^ In comparison to the RMSD distributions obtained from the first 1.0 μs simulations, shown in Figure S2 in the Supporting Information, we observe a notable shift in the distribution for backbone C_α_ and N atoms toward lower values, indicating a closer agreement with experimental data after reaching equilibrium. However, we did not observe a similar trend for C_β_ atoms, where a slight shift toward higher values is noted in comparison to the initial 1.0 μs simulations. δ_sim_ for the C_α_ and C_β_ atoms of Aβ40 fibril display good correlation with δ_exp_ of U-shaped fibrillar model^49^ (see Figure S3 in the Supporting Information), with corresponding *R* values of 0.97 and 0.99, respectively. However, δ_sim_ for the C atoms of the Aβ40 fibril did not demonstrate a very good correlation with the experimental NMR chemical shift of U-shaped Aβ40 fibril, with a corresponding *R* value of 0.77. This discrepancy likely arises from differences in experimental conditions and simulation environments, particularly because the carbonyl carbon (C=O) chemical shift in proteins is influenced by the hydrogen bond network with the surrounding solvent. RMSD distributions, shown in Figure S4 of Supporting Information, display a relatively larger deviation for backbone C_α_ and C atoms, in comparison to the C_β_ atom. It is worth noting that interchain contacts between N- and C-terminal β-sheets involving residue pairs L^17^–L^34^/V^36^, F^19^–L^34^, F^19^/A^21^–I^32^, H^13^–V^40^, and Q^15^–V^36^, which are characteristic of the U-shaped Aβ40 fibrillar model, are frequently observed in our Aβ40 fibril simulations, and discussed in detail in the later section. Additionally, the radius of gyration (*R*_g_) value of the Aβ40 monomer computed from our simulations is 11.7 (±2.6) Å, which qualitatively agrees with the experimental prediction based on hydrodynamic radius measurements using size exclusion chromatography (SEC) and NMR diffusion experiments.^85^ Hence, our simulations employing the CHARMM force-field generate conformational ensembles of Aβ monomer and fibril that qualitatively agree with the experiment and can be used for further study into the effect of TAT protein on Aβ monomer and fibril.

### Effect of TAT Protein on Aβ Monomers

**Figure 2:**
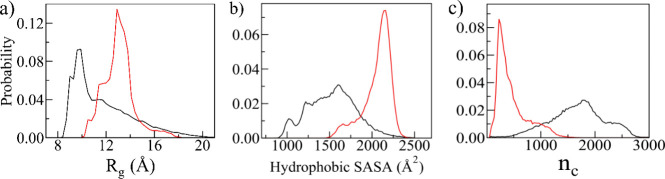
Probability distribution of (a) radius of gyration (*R*_g_), (b) solvent accessible surface area (SASA) of hydrophobic residues, and (c) number of contacts of Aβ monomer in the presence (red) and absence (black) of TAT protein. Data are averaged over the final 2.0 μs of three independent trajectories, for each system.

To examine the influence of the TAT protein on the Aβ monomer, we first computed the Aβ monomer’s radius of gyration (*R*_g_), solvent-accessible surface area (SASA) of hydrophobic residues, and the total number of contacts (*n*_c_) across the simulation trajectories, in both the presence and absence of the TAT protein. As shown in Figure 2, we compared the distributions of *R*_g_, hydrophobic SASA, and *n*_c_ between both systems. The *R*_g_ and hydrophobic SASA distributions are shifted toward the higher value in the presence of TAT protein. The mean *R*_g_ values for the Aβ monomer, averaged over the last 2.0 μs of three independent trajectories, are 11.7 (±2.6) Å in the absence of the TAT protein and 12.9 (±1.3) Å in the presence of the TAT protein. The corresponding mean SASA values for the hydrophobic residues are 1523.7 (±279.4) and 2051.4 (±163.3) Å^2^, respectively. Thus, *R*_g_ and hydrophobic SASA increase by about 10.2 and 34.6% in the presence of the TAT protein. The distribution of *n*_c_ clearly showed a substantial reduction in the total number of contacts within the Aβ monomer, decreasing by about 73% in the presence of the TAT protein. This reduction in contacts is compensated for by the emergence of new interactions with the TAT protein. These analyses provide compelling evidence that the TAT protein induces a shift in the ensembles of Aβ monomers toward extended, more solvent-exposed, and loosely packed conformations.

**Figure 3:**
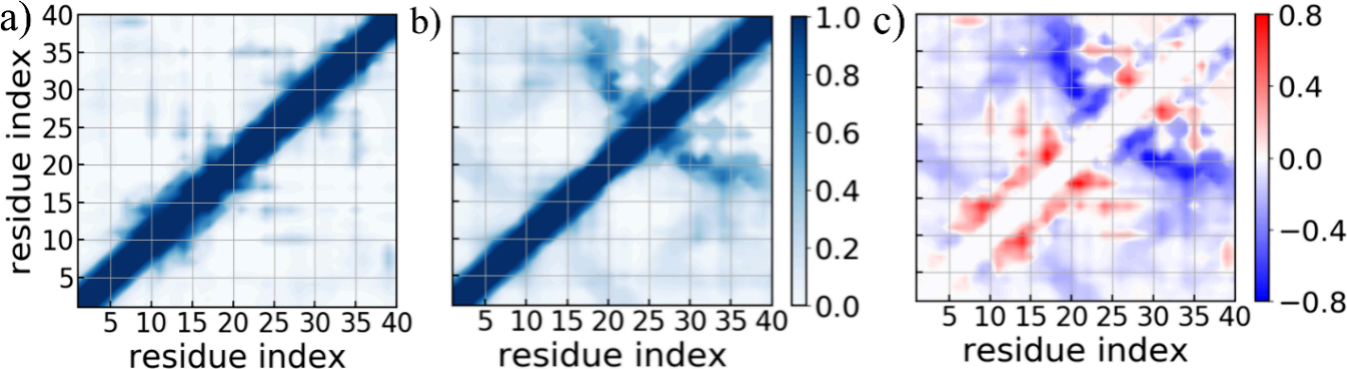
Residue–residue contact frequencies measured in simulations of Aβ monomer in the presence (a) and absence (b) of TAT protein. The differences in contact frequency upon TAT protein binding relative to the control simulation are also shown in (c). Data are averaged over the final 2.0 μs of three independent trajectories, for each system.

**Figure 4:**
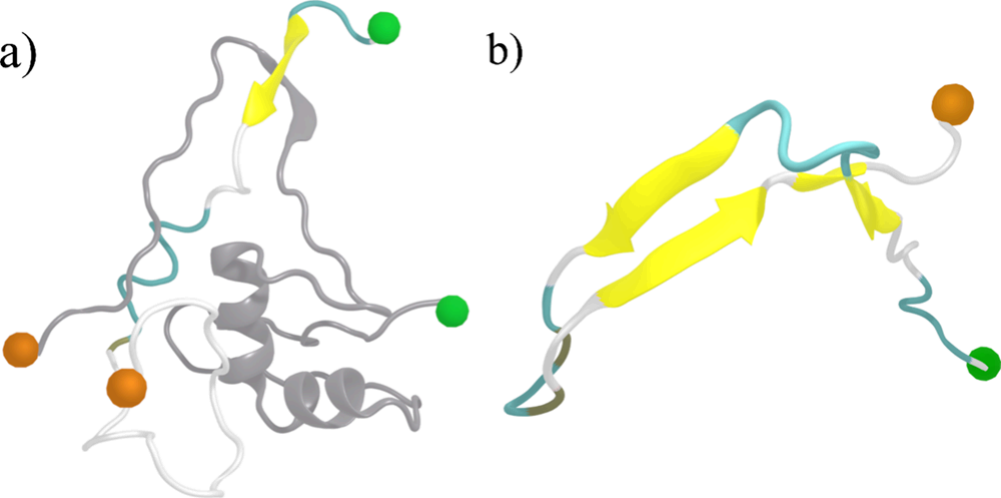
(a) Representative snapshot obtained at the end of 5.0 μs simulations of the Aβ monomer in the presence of TAT protein. (b) Representative snapshot extracted at the end of 4.0 μs control simulation. The TAT protein is colored in gray, and the N- and C-terminal residues are indicated by green and orange spheres, respectively.

Following that, we computed the residue–residue contact probabilities for the Aβ monomer in the presence and absence of TAT protein. The corresponding data are presented in Figure 3a, b, and the differences in residue–residue contact probabilities resulting from the presence of the TAT protein are also shown in Figure 3c. Analyzing contact patterns, we observe a significant reduction in nonlocal contacts between the amphiphilic N-terminus and purely hydrophobic C-terminus, particularly in the hydrophobic contacts between central hydrophobic core (residues 17–21) and C-terminal regions (residues 30–40) in the presence of TAT protein. Numerous prior studies have consistently reported that the Aβ monomer tends to adopt collapse conformations in the aqueous environment, primarily due to the hydrophobic interactions between the central hydrophobic core (CHC) and the C-terminus, which are also observed in this study. As observed in Figure 3, these interactions are significantly disturbed in the presence of the TAT protein. As a consequence, β-sheet propensity within the Aβ monomer, primarily observed in the CHC and C-terminus, decreases from approximately 16% to about 5%. This is also evident in Figure 4a, where we show the representative snapshots obtained at the end of 5.0-μs simulations of Aβ monomer in the presence of TAT protein, and Figure 4b presents the snapshot extracted at the end of 4.0-μs control simulation. We also observed the emergence of an interprotein β-sheet motif between the N-terminus of Aβ, involving residues 5–8 and 56–59 residue of TAT protein (see Figure 4a). Importantly, while the TAT protein decreases β-sheet propensity within the Aβ monomer, it simultaneously increases the solvent exposure of the aggregation-prone regions significantly, thereby increasing the probability of interaction with other Aβ proteins and promoting subsequent aggregation.

To investigate the energetics of the Aβ monomer-TAT protein interaction, we estimated the binding effective energy (Δ*G*_eff_) of the Aβ monomer with the TAT protein using the MM-GBSA protocol as described in the Materials and Methods section. The MM-GBSA method has been widely used in many different areas of biomolecular research particularly in studying protein–ligand binding and protein–protein interactions, and provides a reliable estimate of binding free energy with minimal computational cost. This method also allows us to estimate the contributions of various components to the free energy. The mean and standard deviations of Δ*G*_eff_ and its components are listed in the Supporting Information (Table S5). The binding effective energy data indicate the stronger adhesion between the Aβ monomer and TAT protein. The mean Δ*G*_eff_ value is −138.0 (±33.0) kcal/mol, calculated over the final 2.0 μs of three independent 5.0-μs-long trajectories; the corresponding data for each trajectory is also shown in the Supporting Information (Table S5). Notably, the binding effective energy of the Aβ40 monomer with TAT protein is more negative than the previously reported binding free energy of the Aβ40 dimer,^86,87^ obtained using similar methods. This implies a more favorable interaction between the Aβ40 monomer and TAT protein compared to that between two Aβ40 peptides. Analysis of the various components contributing to Δ*G*_eff_ reveals that the electrostatic interaction (Δ*E*_elec_) between the Aβ monomer and TAT protein is notably stronger than the van der Waals interactions (Δ*E*_vdW_). Nonetheless, Δ*G*_eff_ primarily arises from the van der Waals interactions, as the electrostatic interactions are entirely offset by the large positive value of the polar solvation energy (Δ*G*_solv-pol_), owing to the unfavorable desolvation energy of the polar and charged residues of both peptides. Decomposition of binding effective energy per residue indicates significant contributions from aromatic and hydrophobic residues within Aβ, mostly found in the CHC and C-terminal region, with F^19^, the central residue in the CHC, exhibiting the highest contribution (−5.25 kcal/mol) to the binding effective energy (see Figure S5 in the Supporting Information). The polar and acidic residues contribute relatively less, and positively charged lysine and arginine residues demonstrate minimal unfavorable interactions. In the TAT protein, lysine and arginine residues, particularly those in the basic domain (residues 49–59), exert significant contribution to the binding effective energy, with *R*^55^ exhibiting the highest contribution (−3.67 kcal/mol) (see Figure S6 in the Supporting Information). This implies that the lysine- and arginine-rich basic domain of the TAT protein functions as a crucial binding site for aggregation-prone Aβ monomer. Note that, this domain itself is prone to aggregate, as evident by UV–visible and NMR spectroscopy.^88^ Moreover, hydrophobic residues within the hydrophobic core motif (residues 38–48) also make notable contributions to the binding effective energy. Conversely, residues within the proline-rich and glutamine-rich domains demonstrate minor contributions to the binding effective energy. Importantly, aromatic residues in both peptides have substantial contributions to the binding effective energy, suggesting π–π stacking interactions between the Aβ monomer and TAT protein. Overall, this analysis identifies key residues in both peptides that play a vital role in their interaction.

### Effect of TAT Protein on Aβ Fibrils

**Figure 5:**
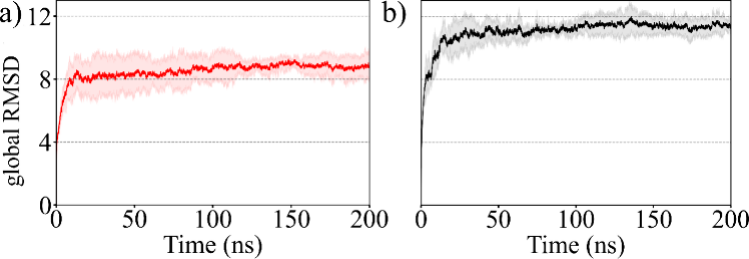
Time evolution of global RMSD (in Å) of Aβ fibril with respect to the experimentally solved structure (PDB ID: 2LMQ) in the presence (a) and absence (b) of the TAT protein. The global RMSD values are averaged over the three independent trajectories for each system, with the shaded region representing the standard deviation.

**Figure 6:**
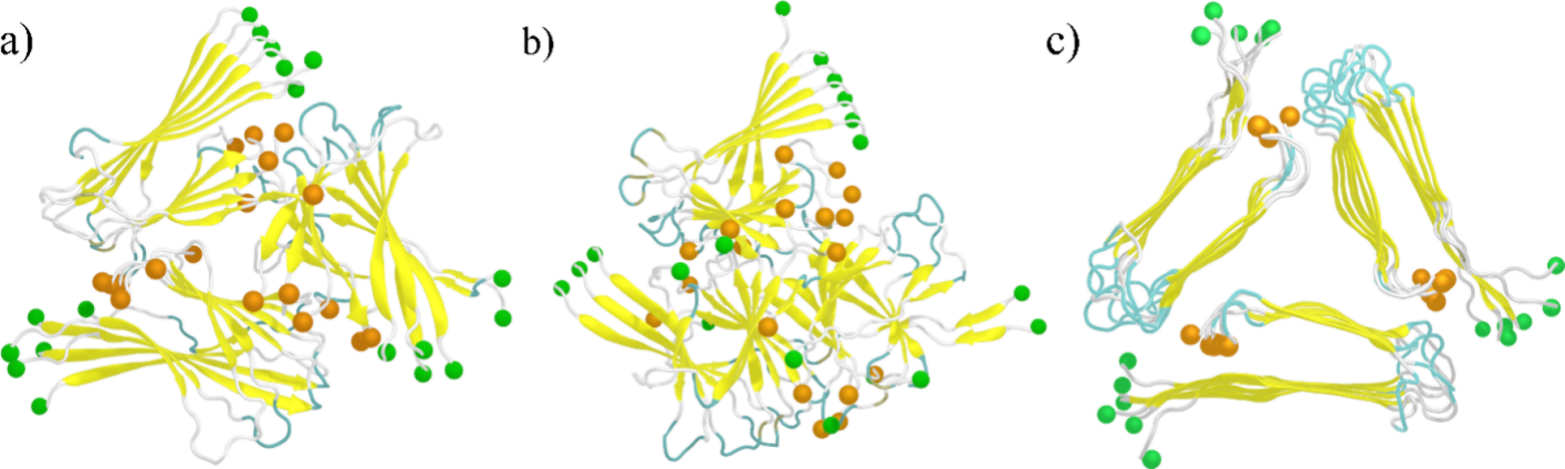
Representative final configuration (at 200 ns) obtained from the simulations of Aβ fibril in the presence (a) and absence (b) of TAT protein. For a comparison, we also show the initial configuration of the Aβ fibril in (c). The N- and C-terminal residues are indicated by green and orange spheres, respectively.

In order to examine the impact of TAT protein on Aβ fibril, we conducted simulations of both Aβ40 fibril and Aβ40 fibril-TAT complex in an aqueous environment. The detailed procedure for generating the starting configuration of the Aβ40 fibril-TAT complex is outlined in the method section. Our analysis began by evaluating the structural stability of the Aβ40 fibril using the metric “local/global RMSD” over the course of the simulation. In Figure 5, we have plotted the time evolution of the global RMSD averaged over three independent trajectories for each system, and compared the data between two cases i.e. in the presence/absence of TAT protein. The global RMSD for the backbone atoms of the fibril is computed after alignment to the relevant NMR structure of the entire fibril (PDB ID: 2LMQ), providing a measure of the structural deviation of the entire fibril. The global RMSD data in both systems reach a plateau within 100 ns time scale, clearly indicating that the simulations attain equilibrium within this time scale. Importantly, the global RMSD value is notably higher in control simulations compared to those in simulations where the TAT protein is bound to the Aβ fibril. The mean global RMSD value, averaged over the final 50 ns and three independent trajectories, is 8.8 (±0.6) Å in the presence of TAT protein, while the corresponding value is 11.4 (±0.5) Å in the absence of TAT protein, suggesting increased stability of the Aβ fibril in the presence of TAT protein. This is also evident by visualizing the final configuration of both systems, shown in Figure 6. Next, we computed the local RMSD and residue-wise RMSF for the backbone atoms of the fibril along the trajectories to measure the local structure change and comprehend the impact of TAT protein on Aβ fibril at a local scale. The corresponding data is presented in Figure S7 in the Supporting Information. Local RMSD was computed after individually aligning them with each relevant protein chain of the NMR structure, providing a measure of the structural distortion for each protein chain within the fibril. Similar to the global RMSD, the local RMSD value is also higher in the control run, although the difference is relatively less pronounced. Examination of the residue-wise backbone RMSF profile revealed increased flexibility in residues near the N- and C-termini, as well as in the U-bent turn regions. Notably, the RMSF of each residue in the Aβ fibril exhibits a greater flexibility in control simulations. Collectively, these analyses provide compelling evidence, indicating the pivotal role of the TAT protein in stabilizing the Aβ fibrillar structure.

To elucidate the factors that contribute to the increased stability of the Aβ fibril in the presence of TAT protein, we computed the total number of heavy atom contacts and hydrogen bonds at the stacking and packing interfaces for both systems with and without TAT protein. The calculation was performed over the final 50 ns of three independent trajectories for each system, and the corresponding mean and standard deviation values are listed in Table 2. Note that the interbackbone (main chain) hydrogen bonds, connecting individual β-strands, play a major role in the stability of the amyloid fibril, contributing approximately 20 times more to providing structural rigidity than the side-chain interactions between β-sheets. Analyzing hydrogen bonding data reveals that the hydrogen bonds between chains primarily comprise interbackbone hydrogen bonds, notably higher at stacking interfaces in the presence of TAT protein. In addition, the amyloid fibril exhibits a significantly higher number of stacking contacts in the presence of the TAT protein. We further examined the stacking contacts at the N-terminal/N-terminal (NN) interface and the C-terminal/C-terminal (CC) interface. Note that the NN interface is stabilized through a mix of hydrophobic and polar interactions, whereas the purely hydrophobic CC interface is stabilized through direct face-to-face interactions between hydrophobic residue side chains. Notably, a significantly higher number of stacking contacts at the NN interface was observed in the presence of the TAT protein, while the increase in stacking contacts at the CC interface was minimal. In contrast, a lower number of contacts and hydrogen bonds were observed at the packing interface in the presence of TAT protein, attributed to the binding of TAT protein at the packing interface (see Figure S8 in the Supporting Information).

**Table 2:** Mean Values of Various Quantities Measured in Simulations of Aβ Fibrils in the Presence/Absence of TAT Protein, Averaged Over the Final 50 ns of Three Independent Trajectories, for Each System^a^

system		stacking hydrogen bond			stacking contact	stacking contact at N-terminus	stacking contact at C-terminus	packing contact	packing hydrogen bond		
		total	backbone–backbone	side chain-side chain					total	backbone–backbone	side chain-side chain
Aβ fibril	control	97.1 (±8.1)	85.3 (±7.2)	5.1 (±2.1)	9981.3 (±301.3)	4725.0 (±160.3)	2883.0 (±87.9)	1339.1 (±233.4)	8.7 (±2.7)	2.2 (±1.4)	1.7 (±1.1)
	with TAT	106.0 (±8.4)	93.9 (±7.8)	5.1 (±2.1)	10,757.7 (±317.1)	5194.9 (±265.1)	2968.5 (±69.2)	864.5 (±120.3)	6.0 (±2.2)	1.2 (±1.0)	0.6 (±0.7)

a Standard deviations are listed within braces.

Since the stability of amyloid fibril is closely correlated with β-sheet content,^89,90^ we measured the total percentage of β-sheet in Aβ fibril for both systems. Importantly, we observed an increase in the total β-sheet content within the Aβ fibril in the presence of the TAT protein. The overall β-sheet percentages within Aβ fibrils were 46.0 and 39.0% in the presence and absence of TAT protein, respectively. This result is in qualitative agreement with previous experimental studies^32,33^ that showed an increase in β-sheet content within Aβ fibrils in the presence of TAT protein. We further point out that the enhancement in the β-sheet is particularly pronounced in the amphiphilic N-terminus, but not in the purely hydrophobic C-terminus. In addition, we have calculated the nematic order parameter (*P*_2_) for Aβ fibril in the presence/absence of TAT protein, following the method^36^ described by Osborne et al., and the corresponding data is shown in Figure S9 in the Supporting Information. *P*_2_ is a commonly used metric for assessing the orientational order of amyloid fibrils,^36,91^ with the *P*_2_ value, ranging from 0 to 1, discriminating between ordered (*P*_2_ = 1) and disordered (*P*_2_ = 0) conformations. As observed in Figure S9, following an initial drop, *P*_2_ reaches a stable value in both systems; however, the value is noticeably higher in the presence of TAT protein. The average *P*_2_ value over the final 50 ns and three independent trajectories is 0.7 (±0.1) in the presence of TAT protein, while the corresponding value is 0.5 (±0.1) in the absence of TAT protein, suggesting that the Aβ fibril adopts a more ordered conformation upon TAT protein binding.

Next, we have estimated the binding effective energy (Δ*G*_eff_) between Aβ fibril and TAT protein using the MM-GBSA protocol, with corresponding data presented in the Supporting Information (Table S8). The absolute value of Δ*G*_eff_ between the Aβ fibril and each TAT protein is almost similar to that of the binding effective energy between the Aβ monomer and TAT protein. Similar to the Aβ monomer-TAT protein complex, Δ*G*_eff_ primarily arises from short-range van der Waals interactions, as the electrostatic interactions are completely canceled out by the large positive value of the polar solvation energy (Δ*G*_solv-pol_). Despite this, we observe a distinct binding pattern. Unlike the Aβ monomer, acidic residues (E^11^, E^22^, and D^23^) in the Aβ fibril contribute significantly to the binding effective energy (see Figure S10 in the Supporting Information). Note that E^22^ and D^23^ are located within the U-bent turn region, positioned adjacent to the packing interface. The hydrophobic and aromatic residues within the Aβ fibril, except V^40^, contribute minimally, and positively charged lysine residues (K^16^ and K^28^) exhibit strong unfavorable interactions. In contrast, within the TAT protein, lysine and arginine residues, particularly those in the basic domain, provide a significant contribution to the effective energy, with K^50^ exhibiting the highest contribution, whereas hydrophobic and aromatic residues contribute relatively less (see Figure S11 in the Supporting Information). This clearly indicates that the aggregation-prone basic domain of TAT protein binds the packing interface of the Aβ fibril through charged interaction with E^22^ and D^23^. As a consequence, contacts within the Aβ fibril at packing interfaces decrease while simultaneously strengthening contacts at stacking interfaces (see Table 2). Figure S8 displays a representative configuration obtained at the end of 200 ns simulation of Aβ fibril in the presence of TAT protein, which also shows that TAT protein binds the Aβ fibril at the packing interfaces. Importantly, our results closely agree with the previous study.^32^

**Figure 7:**
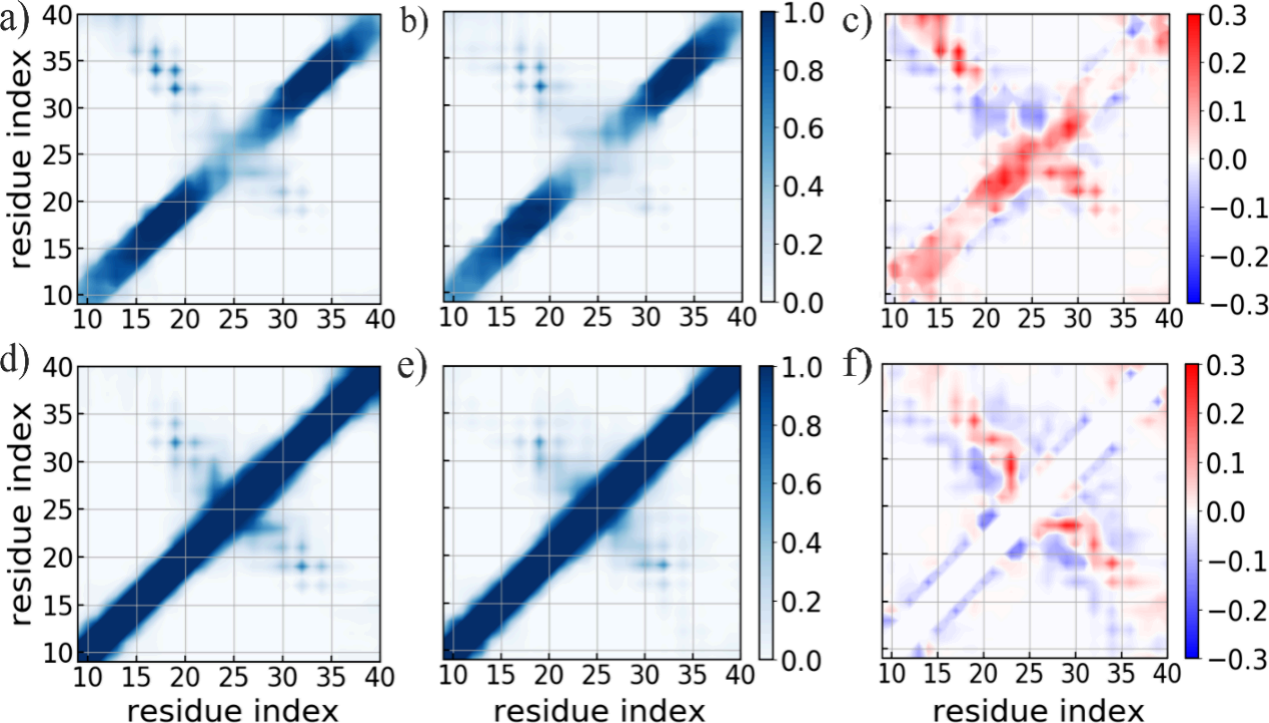
Residue-wise interlayer contact probabilities measured in simulations of Aβ fibril in the presence (a) and absence (b) of the TAT protein. The corresponding intralayer contact probabilities are shown in (d) and (e), respectively. The differences in interlayer and intralayer contact probabilities due to the presence of TAT protein are also shown in (c) and (f), respectively. Data are averaged over the last 50 ns of three independent trajectories, for each system.

We finally compared the tertiary structure of the amyloid fibril with and without the presence of the TAT protein. Figure 7 displays the residue-wise inter/intrachain contact probabilities for the Aβ fibril in the presence/absence of the TAT protein, along with the corresponding differences in contact probabilities. Analyzing the contact pattern, it is evident that the majority of native interchain contacts within the central hydrophobic core remain largely conserved over the simulation in both systems, observed with a frequency of over 80%. Those contacts are seen at a relatively higher frequency in the presence of TAT protein. Additionally, interchain contacts within the non-β strand turn region are increased in the presence of TAT protein but there is no significant difference observed in the interchain contact patterns within the C-terminal region between the two systems. Importantly, interchain contacts between N- and C-terminal β-sheets are significantly enhanced in the presence of TAT protein, thereby stabilizing the staggering of the N- and C-terminal β-sheets. Specifically, the hydrophobic interactions between the central hydrophobic core and the C-terminal region are markedly increased. Note that, interchain contacts between N- and C-terminal β-sheets involving residue pairs L^17^–L^34^/V^36^, F^19^–L^34^, F^19^/A^21^–I^32^, H^13^–V^40^, and Q^15^–V^36^, which are characteristic of the U-shaped Aβ40 fibrillar model, occur at higher frequency in the presence of the TAT protein. We did not find a notable difference in the intrachain contact pattern between the two systems. However, intrachain nonlocal native hydrophobic contacts involving residue pairs L^17^–L^34^, F^19^–L^34^, and A^21^–I^32^, are seen to occur at higher frequency in the presence of TAT protein. In summary, our analysis reveals that the TAT protein stabilizes the contacts between the N- and C-terminal β-sheets, thereby enhancing the β-sheet content within the Aβ fibril.

Additionally, to understand the effect of unresolved N-terminal residues (D1-S8) of the Aβ fibril on TAT protein binding, we have modeled those residues and performed 200 ns simulations in the presence/absence of TAT protein. Disordered N-terminal residues of the Aβ40 fibril are added using CHARMM-GUI PDB Manipulator,^92,93^ followed by subsequent minimization prior to simulation setup. Initial configurations for both systems are shown in Figure S12 in the Supporting Information. Notably, global RMSD data reveal a similar trend to that observed in the Aβ fibril without disordered N-terminus; indicating that the Aβ fibril becomes more stable in the presence of TAT protein (see Figure S13 in the Supporting Information). We next estimated Δ*G*_eff_ between Aβ fibril with disordered N-terminus and TAT protein, with corresponding data presented in Table S11 of the Supporting Information. The binding interactions appear to be relatively more favorable compared to the interactions between the Aβ fibril without the disordered N-terminus and the TAT protein. The mean Δ*G*_eff_ between the Aβ fibril with disordered N-terminus and TAT protein is −180.4 (±26.2) kcal/mol, calculated over the final 50.0 ns of two independent trajectories. The corresponding value between the Aβ fibril without disordered N-terminus and TAT protein is −128.9 (±44.9) kcal/mol. This could potentially be attributed to the strong interaction between the negatively charged residues (D^1^, E^3^, and D^7^) in the disordered N-terminus of the Aβ fibril and the positively charged TAT protein.

## Conclusions

In this study, we have employed explicit-solvent atomistic MD simulation to examine the direct interaction between the HIV-TAT protein and Aβ peptide, which is believed to play a key role in developing AD among HIV-infected individuals.^32,33^ Remarkably, the C_α_ and C_β_ chemical shifts of both the Aβ monomer and fibril, derived from our simulations, exhibit a high correlation with those obtained from prior experimental studies,^49,80^ indicating that the conformational ensemble generated from our simulations employing CHARMM all-atom force-field agree well with experiment. Simulations of the Aβ monomer in the presence of the TAT protein reveal a strong binding affinity between the positively charged TAT protein and the negatively charged Aβ monomer. As a consequence, intramonomer interaction within the Aβ monomer, particularly the nonlocal hydrophobic interactions between the CHC and C-terminal region, is significantly disturbed. This results in increased solvent exposure of these aggregation-prone regions, thereby increasing the likelihood of interaction with other Aβ molecules and facilitating subsequent aggregation. The lysine- and arginine-rich aggregation-prone basic domains, along with the hydrophobic core motif in the TAT protein, were found to play a central role in binding with the Aβ monomer. Note that, prior studies reported the binding free energy of Aβ40 dimer using the same method we’ve employed for our calculations.^86,87^ Nguyen et al. generated equilibrium ensembles of the Aβ40 dimer and its mutants using all-atom replica exchange molecular dynamics (REMD) simulations (24.0 μs per system) and computed binding free energies between two peptide units using molecular mechanics (MM) for the internal energies, alongside three different methods for solvation energies: PBSA, GBSA, and 3DRISM.^86^ The binding free energy value depends exclusively on the method used for computing the solvation energies. The estimated binding free energy of the Aβ40 dimer using the MM-GBSA approach was −70.0 (±22.9) kcal/mol, while using the MM-3DRISM and MM-PBSA methods, the corresponding values were −25.6 (±14.2) and −7.1 (±8.3) kcal/mol, respectively. The entropic contribution was included in the binding free energy calculation, with a total entropic contribution of +43.5 kcal/mol. Watts et al. employed the MM-PBSA approach to compute the binding free energy of the Aβ40 dimer obtained from REMD simulations.^87^ Notably, they did not include the entropic contribution in the binding free energy calculation and the binding free energy was estimated to be −22.4 (±1.5) kcal/mol. Importantly, these values are higher than the binding effective energy value of the Aβ40 monomer with TAT protein, as reported in our study. This suggests a more favorable interaction between the Aβ40 monomer and TAT protein compared to that between two Aβ40 peptides.

Moreover, fibril simulations provided conclusive evidence that the experimentally resolved commonly observed U-shaped Aβ fibril is more stable in the presence of the TAT protein. Notably, the TAT protein exhibits a comparable binding affinity with the Aβ fibril to that with the Aβ monomer. The binding effective energy analysis reveals that the positively charged basic domain of TAT protein binds the packing interface of Aβ fibrils mainly through the charged interaction with E^22^ and D^23^, located within the U-bent turn region near the packing interface. Examination of the contact pattern reveals that nonlocal hydrophobic contacts between N- and C-terminal β-sheets are strengthened in the presence of TAT protein, thereby increasing β-sheet content within Aβ fibril. Importantly, prior experimental studies, employing techniques such as atomic force microscopy (AFM), thioflavin T (ThT) fluorescence, Fourier transform infrared (FT-IR) spectroscopy, and circular dichroism (CD) spectroscopy, have unequivocally demonstrated that TAT protein enhances β-sheet content within Aβ fibril.^32,33^ Our simulations qualitatively agree with these preceding experimental studies,^32,33^ and discern key interactions that stabilize preformed Aβ fibrils and enhance β-sheet content, which may not be discernible through current experimental techniques. Notably, amyloid fibrils display a high degree of polymorphism.^94^ In this study, we specifically examined the effect of the TAT protein on the U-shaped Aβ fibril. Continuing studies by our group will further elucidate this effect on other experimentally resolved Aβ fibrillar structures.

## Data Availability

All methodological details, such as the PDB code and the simulation conditions, are available in the Materials and Methods section. The simulation software and the force field used for running simulations are openly available. Simulation details are listed in Table 1. The visualization software and the software used for analyzing the trajectories are also openly available. The chemical shift data computed from our simulations, which were used to validate the conformation ensemble obtained from our simulations with experimental data, are provided in the Supporting Information (Tables S1–S4). The binding effective energy data are also listed in the Supporting Information (Tables S5–S11).

## Supporting Information Available

Regression plots of calculated and experimentally determined residue-wise chemical shifts; distribution of RMSD between calculated and experimentally determined NMR chemical shifts; binding effective energy between TAT protein and Aβ monomers/fibrils, and per-residue contribution to binding effective energy; local RMSD and RMSF of Aβ fibrils; representative final configuration obtained from the simulations of Aβ fibrils in the presence of TAT protein; time evolution of nematic order parameter (*P*_2_) of Aβ fibrils; starting configuration of Aβ fibril in the presence/absence of TAT protein where the unresolved N-terminal regions (resid 1–8) of Aβ fibril are modeled; and global RMSD of Aβ fibril in the presence/absence of TAT protein where the unresolved N-terminal regions (resid 1–8) of Aβ fibril are modeled (PDF)
