# Supplementary material for: Molecular Insight into the Effect of HIV-TAT Protein on Amyloid-β Peptides
Source: ACS Omega. 2024 Jun 13;9(25):27480–91. doi: 10.1021/acsomega.4c02643 (PMC11209880; doi:10.1021/acsomega.4c02643)
Supplement: Supplementary file 1 — ao4c02643_si_001.pdf [file ao4c02643_si_001.pdf]

Supporting Information

Molecular Insight into the Effect of HIV-TAT Protein on Amyloid -β Peptide

Asis K. Jana*,[a], Recep Keskin[b] and Fatih Yaşar*,[b]

[a]Sister Nivedita University, Department of Microbiology and Biotechnology, Kolkata, INDIA

[b]Hacettepe University, Department of Physics Engineering, Ankara 06800, TÜRKİYE

*Corresponding Authors: asis.kj@snuniv.ac.in, fatih@hacettepe.edu.tr

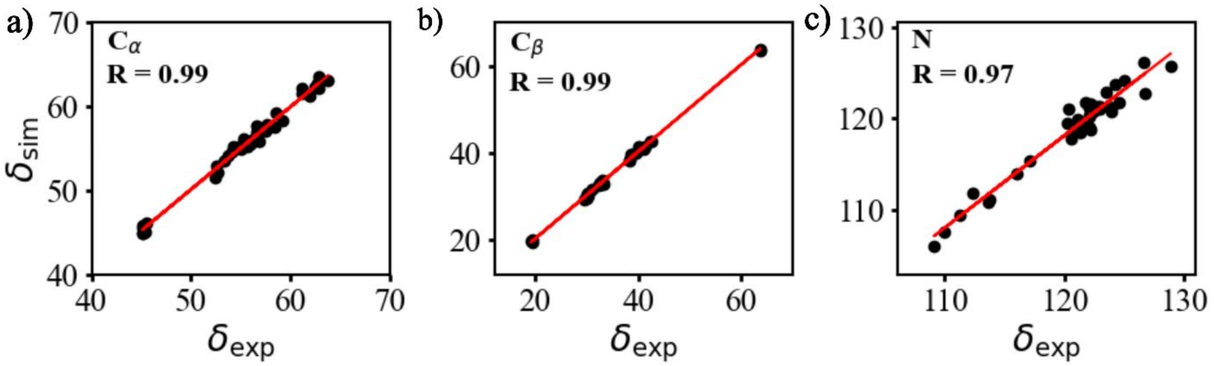

Figure S1: Correlation between calculated (δ_{sim}) and experimentally determined (δ_{exp}) NMR chemical shifts for (a) C_α , (b) C_β and (c) N atoms of the Aβ40 monomer. Linear regression of δ_{sim} and δ_{exp} are shown with the Pearson correlation coefficient (R). The unit of chemical shifts is in ppm.

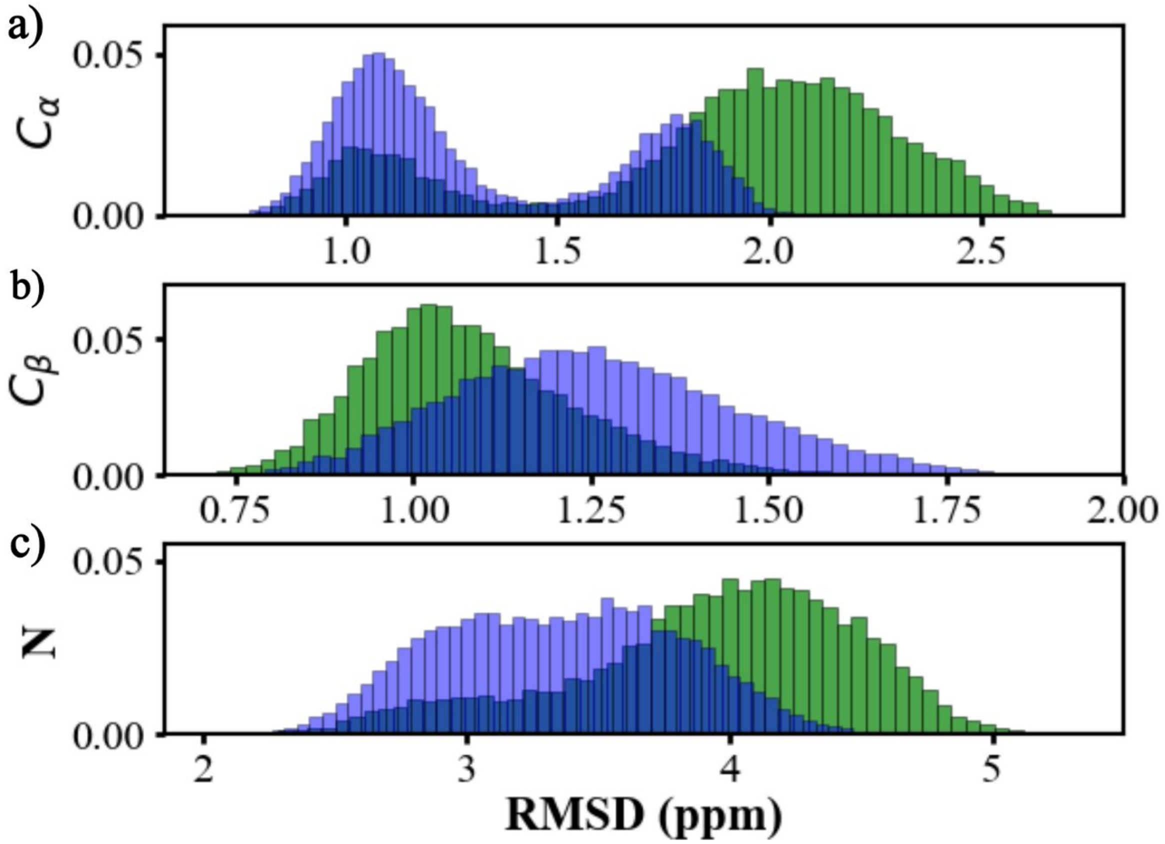

Figure S2: Distribution of root-mean-square-deviation (RMSD) between calculated and experimentally determined NMR chemical shifts for (a) C_α , (b) C_β and (c) N atoms of the Aβ40 monomer. RMSD data obtained from first μs and last 2.0 μs are colored in green and blue, respectively.

Table S1: Calculated and experimentally determined NMR chemical shifts for the C_α and C_β atoms of Aβ40 monomer. The unit of chemical shifts is in ppm.

residue index	residue name	C_α (monomer) calculated	C_α (monomer) experiment	residue index	residue name	C_β (monomer) calculated	C_β (monomer) experiment
1	ASP	54.2	53.7	1	ASP	41.3	40.0
2	ALA	52.1	52.7	2	ALA	19.8	19.1
3	GLU	57.0	56.6	3	GLU	30.7	30.2
4	PHE	57.8	57.6	4	PHE	39.9	39.5
5	ARG	55.2	55.7	5	ARG	31.5	31.0
6	HIS	55.6	55.9	6	HIS	30.3	30.0
7	ASP	54.6	54.1	7	ASP	41.4	41.2
8	SER	58.3	59.2	8	SER	63.9	63.6
9	GLY	45.2	45.4	10	TYR	39.8	38.8
10	TYR	57.6	58.4	11	GLU	30.7	30.3
11	GLU	56.1	56.5	12	VAL	32.9	32.5
12	VAL	62.1	62.8	13	HIS	29.9	29.9
13	HIS	55.8	55.7	14	HIS	29.6	29.9
14	HIS	55.9	55.9	15	GLN	29.4	29.5
15	GLN	55.6	55.9	16	LYS	33.2	33.0
16	LYS	56.2	56.4	17	LEU	42.6	42.3
17	LEU	54.9	55.1	18	VAL	33.6	33.1
18	VAL	61.2	62.0	19	PHE	40.9	40.2
19	PHE	57.1	57.5	20	PHE	40.8	40.1
20	PHE	57.2	57.4	21	ALA	19.5	19.4
21	ALA	51.6	52.4	22	GLU	30.2	30.3
22	GLU	57.7	56.6	23	ASP	41.0	41.1
23	ASP	55.2	54.2	24	VAL	32.5	32.3
24	VAL	63.5	62.8	26	SER	63.5	63.7
25	GLY	46.2	45.5	27	ASN	39.7	38.5
26	SER	59.2	58.6	28	LYS	33.2	32.5
27	ASN	53.5	53.3	30	ALA	19.9	19.3
28	LYS	55.9	56.8	31	ILE	38.2	38.4
29	GLY	45.0	45.2	32	ILE	38.4	38.4
30	ALA	53.0	52.5	34	LEU	42.7	42.5
31	ILE	62.2	61.1	35	MET	32.9	32.6
32	ILE	61.6	61.2	36	VAL	32.7	32.7
33	GLY	45.8	45.2	39	VAL	32.7	32.9
34	LEU	55.6	55.2	40	VAL	32.9	33.2
35	MET	56.1	55.3				
36	VAL	62.8	62.7				
37	GLY	45.6	45.2				
38	GLY	45.0	45.1				
39	VAL	62.3	62.5				
40	VAL	63.1	63.8				

Table S2: Calculated and experimentally determined NMR chemical shifts for the backbone N atoms of Aβ40 monomer. The unit of chemical shifts is in ppm.

residue index	residue name	N (monomer) calculated	N (monomer) Experiment
2	ALA	123.8	124.3
3	GLU	118.8	121.0
4	PHE	121.2	122.1
5	ARG	121.1	123.9
6	HIS	119.4	121.4
7	ASP	120.2	122.1
8	SER	115.4	117.1
9	GLY	109.5	111.2
10	TYR	117.8	120.6
11	GLU	121.3	123.0
12	VAL	121.8	121.8
13	HIS	121.4	122.7
14	HIS	118.5	121.3
15	GLN	119.1	122.1
16	LYS	120.8	123.9
17	LEU	121.7	124.6
18	VAL	120.4	122.1
19	PHE	124.2	125.0
20	PHE	122.9	123.5
21	ALA	126.1	126.7
22	GLU	121.0	120.4
23	ASP	121.6	122.2
24	VAL	119.9	121.1
25	GLY	111.9	112.3
26	SER	114.0	116.0
27	ASN	119.3	121.0
28	LYS	118.9	122.2
29	GLY	107.6	110.0
30	ALA	121.2	124.0
31	ILE	118.5	121.3
32	ILE	122.8	126.8
33	GLY	110.8	113.6
34	LEU	121.2	122.1
35	MET	120.8	122.4
36	VAL	121.0	123.0
37	GLY	111.1	113.7
38	GLY	106.0	109.1
39	VAL	119.5	120.3
40	VAL	125.7	128.9

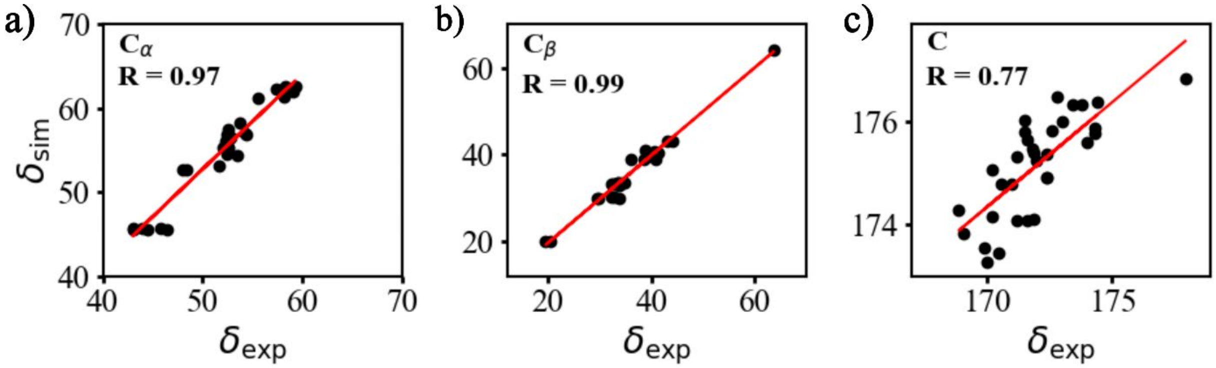

Figure S3: Correlation between calculated (δ_{sim}) and experimentally determined (δ_{exp}) NMR chemical shifts for (a) C_α , (b) C_β and (c) C atoms of the Aβ40 fibril. Linear regression of δ_{sim} and δ_{exp} are shown with the Pearson correlation coefficient (R). The unit of chemical shifts is in ppm.

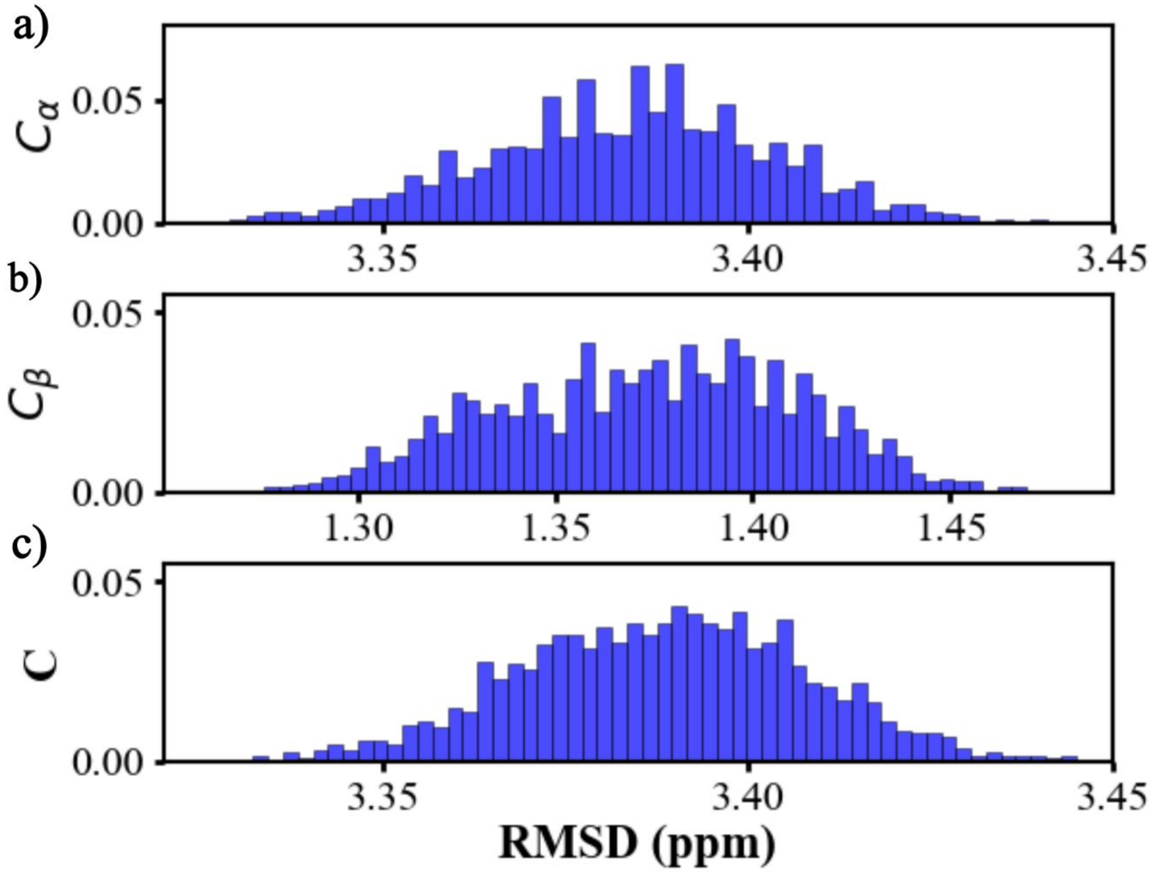

Figure S4: Distribution of root-mean-square-deviation (RMSD) between calculated and experimentally determined NMR chemical shifts for (a) C_α , (b) C_β and (c) C atoms of the Aβ40 fibril.

Table S3: Calculated and experimentally determined NMR chemical shifts for the C_α and C_β atoms of Aβ40 fibril. The unit of chemical shifts is in ppm.

residue index	residue name	C_α (fibril) calculated	C_α (fibril) experiment	residue index	residue name	C_β (fibril) calculated	C_β (fibril) experiment
9	GLY	45.6	44.5	10	TYR	39.0	36.0
10	TYR	57.5	52.5	11	GLU	30.0	29.7
11	GLU	56.5	52.8	12	VAL	33.1	33.7
12	VAL	62.6	59.3	13	HIS	30.0	29.5
13	HIS	55.3	52.5	14	HIS	30.0	33.7
14	HIS	55.3	52.0	15	GLN	30.2	32.8
15	GLN	56.1	52.3	16	LYS	33.6	34.3
16	LYS	56.5	52.6	17	LEU	43.2	43.2
17	LEU	55.2	52.2	18	VAL	33.5	33.6
18	VAL	62.2	58.9	19	PHE	40.6	40.5
19	PHE	56.9	54.4	20	PHE	40.4	41.2
20	PHE	57.0	54.3	21	ALA	20.0	20.4
21	ALA	52.6	48.0	22	GLU	30.2	32.2
22	GLU	56.5	52.7	23	ASP	40.9	38.8
23	ASP	54.3	53.5	24	VAL	33.1	32.4
24	VAL	62.5	58.3	26	SER	64.2	63.7
25	GLY	45.7	44.0	27	ASN	39.4	39.0
26	SER	58.3	53.8	28	LYS	33.5	33.6
27	ASN	53.2	51.6	30	ALA	20.1	19.5
28	LYS	56.9	52.4	31	ILE	38.9	38.5
29	GLY	45.6	43.0	32	ILE	39.0	40.7
30	ALA	52.7	48.4	34	LEU	43.2	44.1
31	ILE	61.4	58.2	35	MET	33.5	34.7
32	ILE	61.2	55.5	36	VAL	33.2	32.9
33	GLY	45.6	46.4	39	VAL	33.3	32.2
34	LEU	55.3	52.5	40	VAL	33.1	33.2
35	MET	54.5	52.4				
36	VAL	62.4	57.4				
37	GLY	45.6	45.8				
38	GLY	45.6	43.0				
39	VAL	62.0	59.1				
40	VAL	62.3	59.0				

Table S4: Calculated and experimentally determined NMR chemical shifts for the C of Aβ40 fibril. The unit of chemical shifts is in ppm.

residue index	residue name	C (fibril) calculated	C (fibril) experiment
9	GLY	173.4	170.5
10	TYR	175.2	172.0
11	GLU	175.8	171.5
12	VAL	175.4	172.4
13	HIS	174.1	171.2
14	HIS	174.2	170.2
15	GLN	175.1	170.2
16	LYS	175.5	171.8
17	LEU	176.0	173.0
18	VAL	174.9	172.4
19	PHE	174.8	171.0
20	PHE	174.8	170.6
21	ALA	176.3	173.4
22	GLU	175.9	174.3
23	ASP	175.8	174.3
24	VAL	176.4	174.4
25	GLY	174.1	171.6
26	SER	174.1	171.9
27	ASN	174.9	172.4
28	LYS	176.3	173.8
29	GLY	173.5	169.9
30	ALA	176.5	172.8
31	ILE	175.4	171.9
32	ILE	175.6	174.0
33	GLY	173.3	170.0
34	LEU	176.0	171.5
35	MET	175.3	171.2
36	VAL	175.8	172.6
37	GLY	174.3	168.9
38	GLY	173.8	169.1
39	VAL	175.7	171.6
40	VAL	176.9	177.9

Table S5: Binding effective energy (ΔG_{eff}) of Aβ monomer with TAT protein for the three trajectories. Data are averaged over the final 2.0 μs of three independent trajectories. Standard deviations are presented within braces. Values are reported in unit of kcal/mol.

Aβ monomer-TAT complex	ΔE_{elec}	ΔE_{vdW}	$\Delta G_{sol\text{-}pol}$	$\Delta G_{sol\text{-}np}$	ΔH_{MM}	ΔG_{solv}	ΔG_{eff}
run 1	-1129.7(130.0)	-144.9(14.4)	1145.0(123.0)	-20.4(1.9)	-1274.5(132.7)	1124.6(122.0)	-149.9(17.1)
run 2	-1184.1(101.6)	-166.9(14.4)	1216.8(94.4)	-26.2(1.9)	-1351.0(103.7)	1190.6(93.5)	-160.4(17.4)
run 3	-925.7(152.0)	-116.5(26.9)	956.2(150.3)	-17.5(3.9)	-1042.3(169.4)	938.7(147.5)	-103.6(28.9)
mean	-1079.8(170.7)	-142.8(28.4)	1106.0(166.2)	-21.4(4.5)	-1222.6(190.4)	1084.6(162.8)	-138.0(33.0)

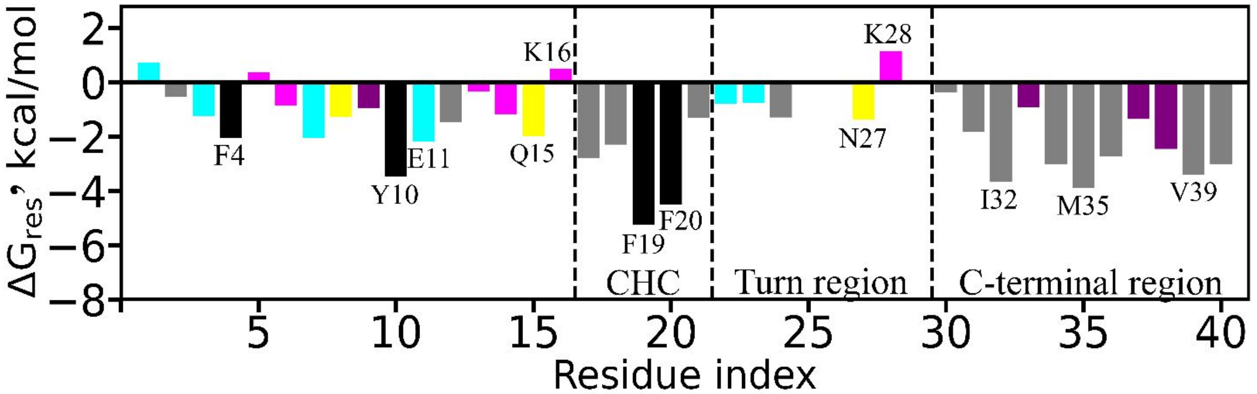

Figure S5: Residue-wise binding effective energy (ΔG_{res}') decomposition of Aβ monomer in Aβ monomer-TAT complex. Data are averaged over the final 2.0 μs of three independent trajectories. The aromatic, hydrophobic, acidic, basic and polar residues are colored in black, grey, cyan, magenta, and yellow, respectively.

Table S6: Residue-wise binding effective energy of Aβ monomer in Aβ monomer-TAT complex. Values are reported in unit of kcal/mol.

residue index	residue name	run1	run2	run3	mean
1	ASP	0.6	0.7	0.8	0.7
2	ALA	-0.3	-0.8	-0.5	-0.5
3	GLU	-1.0	-1.6	-1.2	-1.2
4	PHE	-0.8	-3.2	-2.1	-2.0
5	ARG	0.5	0.4	0.1	0.4
6	HIS	-0.2	-0.6	-1.6	-0.8
7	ASP	-1.3	-2.0	-2.8	-2.1
8	SER	0.1	-1.8	-2.1	-1.3
9	GLY	-0.1	-1.1	-1.7	-0.9
10	TYR	-0.6	-6.4	-3.4	-3.5
11	GLU	-2.4	-2.9	-1.3	-2.2
12	VAL	-1.3	-1.2	-1.9	-1.5
13	HIS	0.0	-0.2	-0.9	-0.3
14	HIS	-1.6	-1.5	-0.4	-1.2
15	GLN	-3.2	-2.7	-0.1	-2.0
16	LYS	-0.2	1.2	0.5	0.5
17	LEU	-2.3	-3.9	-2.1	-2.8
18	VAL	-4.5	-2.0	-0.3	-2.3
19	PHE	-10.6	-4.6	-0.6	-5.2
20	PHE	-10.1	-0.3	-3.1	-4.5
21	ALA	-2.5	-1.0	-0.4	-1.3
22	GLU	-1.4	-0.7	-0.3	-0.8
23	ASP	-0.7	-1.0	-0.5	-0.8
24	VAL	-0.7	-2.8	-0.5	1.3
25	GLY	0.2	-0.2	-0.1	-0.0
26	SER	0.2	-0.3	0.0	-0.1
27	ASN	-0.2	-3.8	-0.2	-1.4
28	LYS	1.2	1.3	0.9	1.1
29	GLY	0.0	-0.1	-0.2	-0.1
30	ALA	0.0	-0.4	-0.8	-0.4
31	ILE	-1.1	-2.4	-2.0	-1.8
32	ILE	-5.0	-3.8	-2.2	-3.7
33	GLY	-2.0	-0.2	-0.5	-0.9
34	LEU	-4.7	-1.9	-2.5	-3.0
35	MET	-3.1	-5.4	-3.2	-3.9
36	VAL	-1.1	-4.8	-2.3	-2.7
37	GLY	-0.7	-2.1	-1.3	-1.4
38	GLY	-2.5	-3.8	-1.0	-2.5
39	VAL	-4.3	-3.6	-2.3	-3.4
40	VAL	-2.5	-3.5	-3.1	-3.0

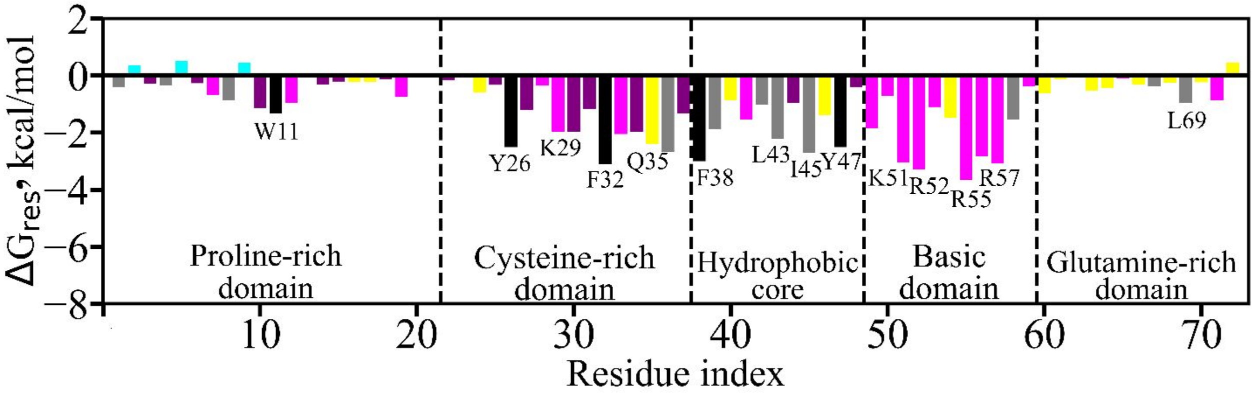

Figure S6: Residue-wise binding effective energy (ΔG_{res}') decomposition of TAT protein in Aβ monomer-TAT complex. Data are averaged over the final 2.0 μs of three independent trajectories. The aromatic, hydrophobic, acidic, basic and polar residues are colored in black, grey, cyan, magenta, and yellow, respectively.

Table S7: Residue-wise binding effective energy of TAT protein in Aβ monomer-TAT complex. Values are reported in unit of kcal/mol.

residue index	residue name	run1	run2	run3	mean
1	MET	-0.2	-0.6	-0.4	-0.4
2	GLU	0.4	0.4	0.3	0.4
3	PRO	-0.1	-0.6	-0.2	-0.3
4	VAL	-0.2	-0.6	-0.2	-0.3
5	ASP	0.5	0.5	0.5	0.5
6	PRO	-0.2	-0.4	-0.2	-0.3
7	ARG	-0.3	-1.5	-0.3	-0.7
8	LEU	-0.3	-1.7	-0.6	-0.9
9	GLU	0.6	0.4	0.3	0.4
10	PRO	-1.1	-1.8	-0.5	-1.1
11	TRP	-2.1	-0.9	-1.0	-1.3
12	LYS	-0.5	-2.2	-0.2	-1.0
13	HIS	0.0	-0.2	-0.1	-0.1
14	PRO	-0.2	-0.5	-0.2	-0.3
15	GLY	-0.2	-0.5	0.0	-0.2
16	SER	-0.6	-0.1	0.0	-0.2
17	GLN	-0.2	-0.5	0.0	-0.2
18	PRO	-0.3	0.0	-0.1	-0.1
19	LYS	-1.4	-0.7	-0.2	-0.7
20	THR	-0.1	0.0	-0.1	-0.1
21	ALA	0.0	0.0	-0.1	0.0
22	CYS	-0.2	0.1	-0.4	-0.2
23	THR	0.0	0.0	0.0	0.0
24	ASN	0.0	-1.6	-0.1	-0.6
25	CYS	-0.4	-0.4	-0.2	-0.3
26	TYR	0.0	-5.0	-2.6	-2.5
27	CYS	-0.2	-2.9	-0.6	-1.2
28	LYS	-0.7	-0.1	-0.3	-0.4
29	LYS	-4.4	-0.6	-0.9	-2.0
30	CYS	-2.6	-2.3	-1.1	-2.0
31	CYS	-0.9	-2.1	-0.5	-1.2
32	PHE	-4.1	-4.8	-0.5	-3.1
33	HIS	-5.2	-0.7	-0.2	-2.1
34	CYS	-4.3	-1.4	-0.2	-2.0
35	GLN	-5.0	-1.2	-1.0	-2.4
36	VAL	-4.1	-2.4	-1.5	-2.7
37	CYS	-1.6	-2.2	-0.2	-1.3
38	PHE	-3.3	-4.6	-1.1	-3.0
39	ILE	-0.5	-2.4	-2.7	-1.9

40	THR	0.0	-1.9	-0.7	-0.9
41	LYS	-0.2	-4.1	-0.4	-1.6
42	ALA	0.0	-1.0	-2.1	-1.0
43	LEU	-1.4	-2.1	-3.2	-2.2
44	GLY	-1.9	-0.5	-0.5	-1.0
45	ILE	-3.9	-1.8	-2.5	-2.7
46	SER	-1.8	-1.4	-0.9	-1.4
47	TYR	-0.8	-4.3	-2.4	-2.5
48	GLY	-0.4	0.1	-0.9	-0.4
49	ARG	-0.8	-1.7	-3.1	-1.9
50	LYS	-0.7	-0.3	-1.1	-0.7
51	LYS	-0.3	-7.0	-1.8	-3.0
52	ARG	-7.7	-0.7	-1.4	-3.3
53	ARG	-0.4	-1.2	-1.7	-1.1
54	GLN	0.1	-3.7	-0.8	-1.5
55	ARG	-5.2	-0.7	-5.1	-3.7
56	ARG	-2.6	-2.1	-3.8	-2.8
57	ARG	-4.4	-2.3	-2.6	-3.1
58	ALA	-3.3	0.1	-1.3	-1.5
59	HIS	-1.0	0.2	-0.3	-0.4
60	GLN	-1.0	-0.2	-0.8	-0.6
61	ASN	0.1	-0.1	-0.3	-0.1
62	SER	0.0	0.0	-0.3	-0.1
63	GLN	0.0	-1.2	-0.4	-0.5
64	THR	0.0	-0.8	-0.5	-0.4
65	HIS	0.1	-0.1	-0.3	-0.1
66	GLN	0.0	-0.2	-0.8	-0.3
67	ALA	0.0	-0.7	-0.4	-0.4
68	SER	-0.2	-0.4	-0.2	-0.3
69	LEU	-0.5	-1.3	-1.0	-1.0
70	SER	-0.1	-0.4	-0.3	-0.2
71	LYS	-0.5	-1.2	-1.0	-0.9
72	GLN	0.4	0.7	0.3	0.4

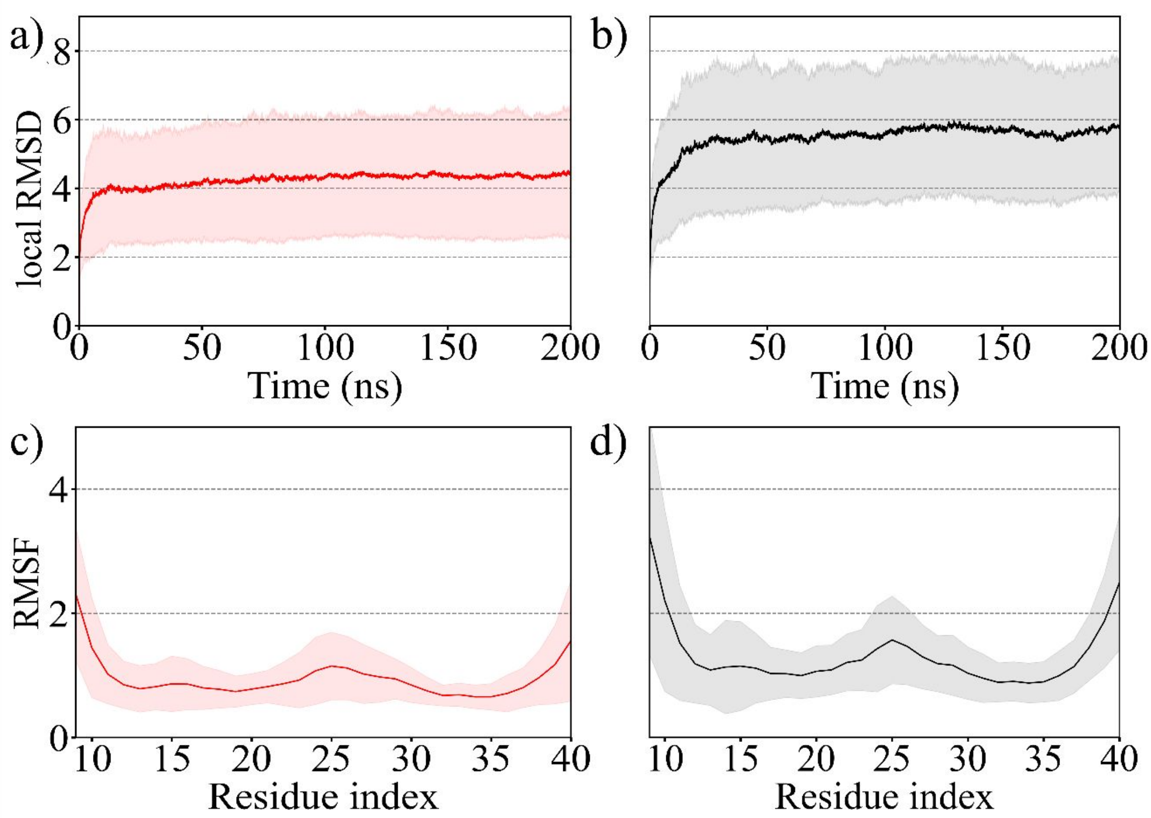

Figure S7: Time evolution of local RMSD (in Å) of Aβ fibril in the presence (a) and absence (b) of TAT protein. The local RMSD values are averaged across all chains in the Aβ fibril and all three trajectories for each system, with the shaded region representing the standard deviation. Residue-wise backbone RMSF (in Å) of Aβ fibril in the presence (c) and absence (d) of TAT protein. The RMSF values are averaged across all chains in the Aβ fibril and the final 50 ns of three trajectories for each system, with the shaded region representing the standard deviation.

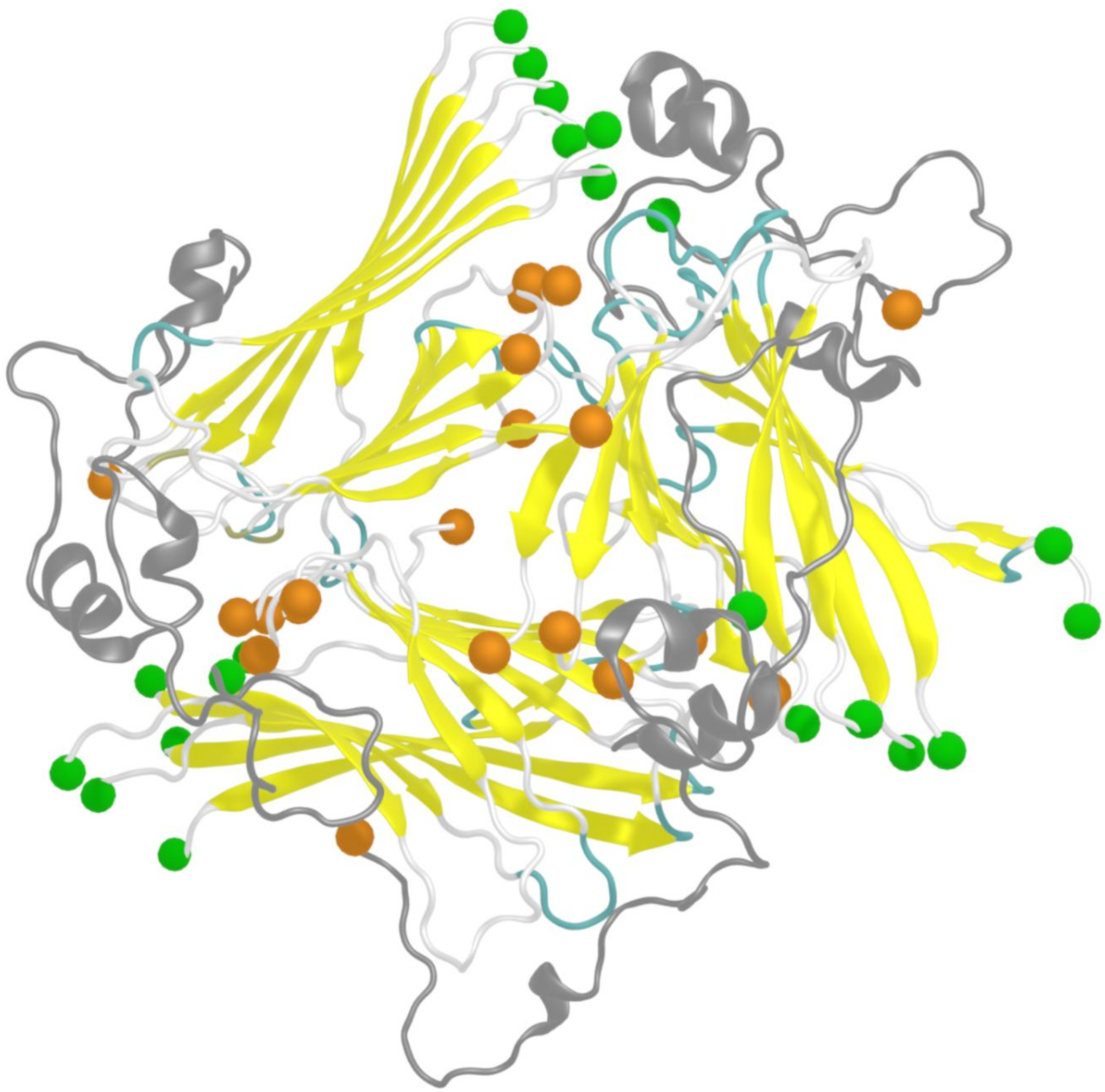

Figure S8: Representative final configuration obtained from the simulations of Aβ fibril in the presence of TAT protein. TAT proteins are colored in grey, and the N- and C-terminal residues are indicated by green and orange spheres, respectively.

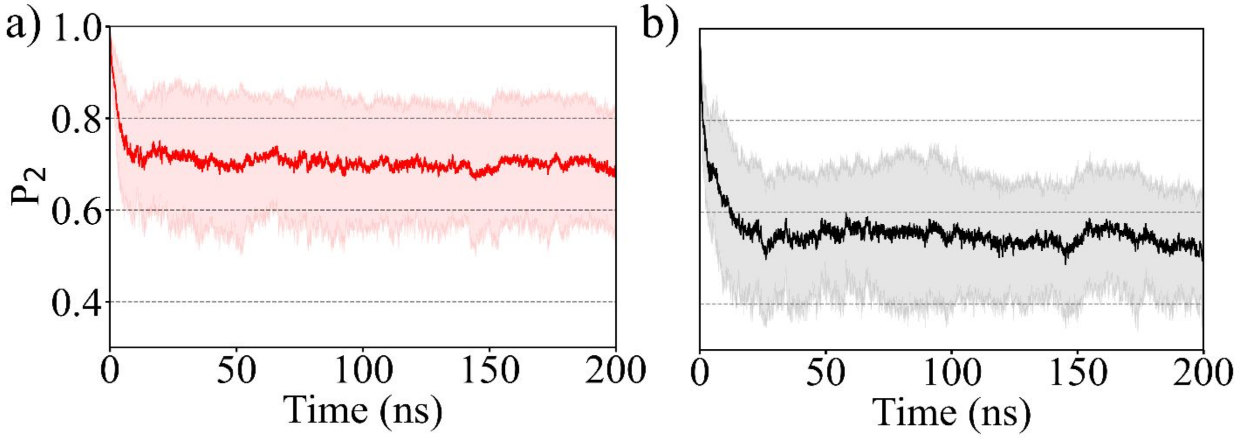

Figure S9: Time evolution of nematic order parameter (P_2) of Aβ fibril in the presence (a) and absence (b) of TAT protein. Data are averaged over the three independent trajectories for each system, with the shaded region representing the standard deviation.

Table S8: Binding effective energy (ΔG_{eff}) of Aβ fibril with each TAT protein for the three trajectories. Data are averaged over the final 50.0 ns of three independent trajectories. Standard deviations are presented within braces. Values are reported in unit of kcal/mol.

Aβ fibril-TAT complex	ΔE_{elec}	ΔE_{vdW}	$\Delta G_{sol\text{-}pol}$	$\Delta G_{sol\text{-}np}$	ΔH_{MM}	ΔG_{solv}	ΔG_{eff}
run 1	-2922.8(659.4)	-107.8(34.0)	2935.2(631.7)	-19.4(7.6)	-3030.6(679.6)	2915.8(626.0)	-114.8(62.7)
run 2	-3513.8(335.1)	-119.5(39.1)	3521.1(324.0)	-21.1(5.0)	-3633.3(309.8)	3500.0(327.1)	-133.3(33.0)
run 3	-3242.1(218.6)	-133.4(33.1)	3260.1(224.6)	-23.3(4.4)	-3375.5(243.3)	3236.8(221.0)	-138.6(26.8)
mean	-3226.3(506.5)	-120.2(37.0)	3238.8(492.2)	-21.2(6.0)	-3346.5(516.4)	3217.6(489.5)	-128.9(44.9)

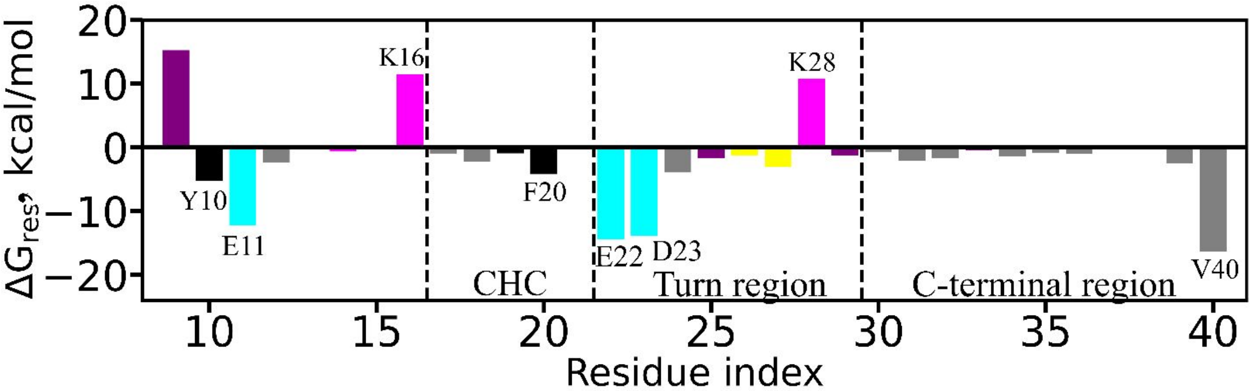

Figure S10: Residue-wise binding effective energy (ΔG_{res}') decomposition of Aβ fibril in Aβ fibril-TAT complex. Data are averaged over the final 50.0 ns of three independent trajectories. The aromatic, hydrophobic, acidic, basic and polar residues are colored in black, grey, cyan, magenta, and yellow, respectively.

Table S9: Residue-wise binding effective energy of Aβ fibril in Aβ fibril-TAT complex. Values are reported in unit of kcal/mol.

residue index	residue name	run1	run2	run3	mean
9	GLY	17.1	13.9	14.8	15.2
10	TYR	-5.4	-2.7	-7.7	-5.3
11	GLU	-14.9	-10.3	-11.5	-12.3
12	VAL	-2.0	-1.8	-3.4	-2.4
13	HIS	-0.2	-0.5	0.7	0.0
14	HIS	-0.4	-2.0	0.4	-0.6
15	GLN	-0.9	0	0.2	-0.2
16	LYS	12.1	11.0	11.3	11.5
17	LEU	-0.9	-0.7	-1.3	-0.9
18	VAL	-1.4	-2.7	-2.7	-2.2
19	PHE	-1.4	-0.7	-0.7	-0.9
20	PHE	-2.5	-5.8	-4.3	-4.2
21	ALA	-0.2	0.4	-0.2	0.0
22	GLU	-12.2	-14.8	-16.4	-14.4
23	ASP	-11.3	-16.4	-13.9	-13.9
24	VAL	-2.0	-5.6	-4.3	-3.9
25	GLY	-0.7	-2.3	-1.8	-1.7
26	SER	-0.9	-1.3	-1.8	-1.3
27	ASN	-1.3	-2.3	-5.8	-3.1
28	LYS	10.1	11.2	11.0	10.8
29	GLY	-0.5	-2.7	-0.5	-1.2
30	ALA	-0.5	-1.3	-0.2	-0.7
31	ILE	-4.1	-1.4	-0.5	-2.1
32	ILE	-1.8	-1.3	-2.0	-1.7
33	GLY	-1.8	0.0	0.2	-0.5
34	LEU	-1.1	-1.1	-2.2	-1.4
35	MET	-2.3	0.0	-0.2	-0.8
36	VAL	-0.2	-0.4	-2.7	-1.1
37	GLY	0.2	0.2	-0.7	-0.1
38	GLY	0.2	0.2	-0.5	-0.1
39	VAL	-2.3	-2.0	-3.2	-2.5
40	VAL	-17.6	-14.0	-17.3	-16.3

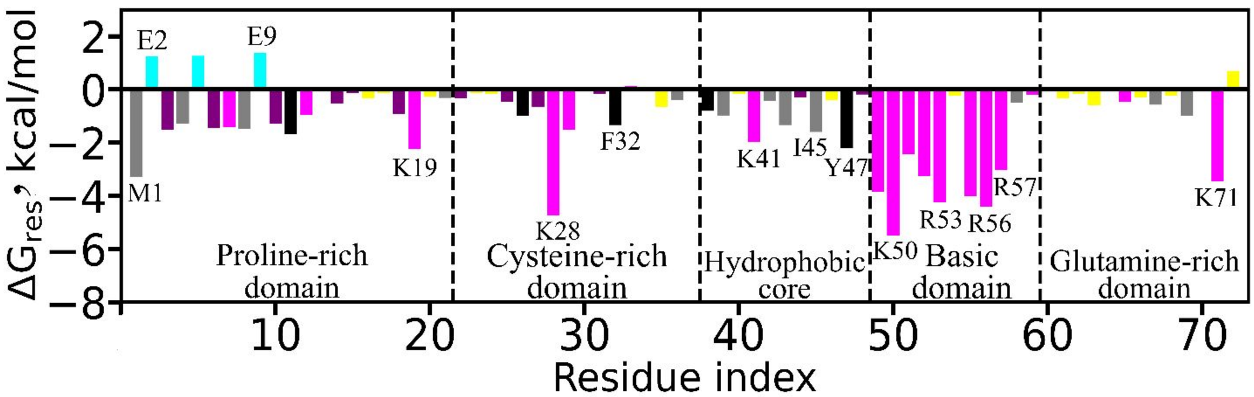

Figure S11: Residue-wise binding effective energy (ΔG_{res}') decomposition of TAT protein in Aβ fibril-TAT complex. Data are averaged over the final 50.0 ns of three independent trajectories. The aromatic, hydrophobic, acidic, basic and polar residues are colored in black, grey, cyan, magenta, and yellow, respectively.

Table S10: Residue-wise binding effective energy of TAT protein in Aβ fibril-TAT complex. Values are reported in unit of kcal/mol.

residue index	residue name	run1	run2	run3	mean
1	MET	-2.8	-4.4	-2.6	-3.3
2	GLU	1.4	0.8	1.5	1.2
3	PRO	-0.7	-0.9	-2.9	-1.5
4	VAL	-0.2	-2.0	-1.7	-1.3
5	ASP	0.5	1.9	1.4	1.3
6	PRO	-1.2	-1.7	-1.5	-1.5
7	ARG	-1.0	-1.4	-1.8	-1.4
8	LEU	-0.9	-3.0	-0.6	-1.5
9	GLU	1.4	1.1	1.6	1.4
10	PRO	-0.9	-1.8	-1.2	-1.3
11	TRP	-2.5	-0.9	-1.7	-1.7
12	LYS	-1.8	-0.2	-0.9	-1.0
13	HIS	-0.4	0.3	0.0	0.0
14	PRO	-0.8	-0.7	-0.1	-0.5
15	GLY	-0.4	0.0	0.0	-0.1
16	SER	-1.2	0.1	0.1	-0.3
17	GLN	0.1	0.0	-0.5	-0.1
18	PRO	-2.0	0.0	-0.8	-0.9
19	LYS	-3.0	-0.7	-3.0	-2.3
20	THR	-0.7	0.0	-0.1	-0.3
21	ALA	-0.9	0.1	-0.1	-0.3
22	CYS	-0.7	0.0	-0.3	-0.3
23	THR	-0.5	0.1	0.1	-0.1
24	ASN	-0.4	-0.1	-0.0	-0.2
25	CYS	-0.4	-0.0	-1.0	-0.5
26	TYR	-0.2	-1.0	-1.7	-1.0
27	CYS	0.0	-1.4	-0.6	-0.7
28	LYS	-1.4	-7.4	-5.4	-4.7
29	LYS	-0.9	-2.6	-1.1	-1.5
30	CYS	0.0	-0.1	-0.2	-0.1
31	CYS	0.0	-0.3	-0.2	-0.2
32	PHE	0.0	-3.1	-1.0	-1.3
33	HIS	0.1	0.2	0.1	0.1
34	CYS	-0.1	0.1	-0.1	-0.0
35	GLN	-0.2	-1.3	-0.5	-0.7
36	VAL	-0.4	-0.6	-0.2	-0.4
37	CYS	-0.1	-0.1	-0.1	-0.1
38	PHE	-0.2	-0.8	-1.4	-0.8
39	ILE	-0.6	-1.2	-1.2	-1.0
40	THR	-0.6	0.1	-0.1	-0.2

41	LYS	-3.3	-1.1	-1.6	-2.0
42	ALA	-0.2	-0.5	-0.6	-0.4
43	LEU	-0.9	-0.9	-2.2	-1.3
44	GLY	-0.3	-0.5	-0.1	-0.3
45	ILE	-1.7	-1.0	-2.1	-1.6
46	SER	-0.8	-0.6	0.1	-0.4
47	TYR	-3.2	-2.0	-1.5	-2.2
48	GLY	-0.1	0.0	-0.6	-0.2
49	ARG	-3.9	-3.6	-4.1	-3.9
50	LYS	-6.8	-2.6	-7.0	-5.5
51	LYS	-3.0	-2.2	-2.1	-2.4
52	ARG	-1.5	-5.8	-2.4	-3.3
53	ARG	-8.0	-1.2	-3.6	-4.3
54	GLN	0.2	-0.7	-0.2	-0.2
55	ARG	-1.5	-6.9	-3.7	-4.0
56	ARG	-2.6	-7.9	-2.7	-4.4
57	ARG	-1.7	-5.3	-2.2	-3.0
58	ALA	0.0	-0.8	-0.7	-0.5
59	HIS	0.3	-1.1	0.2	-0.2
60	GLN	0.2	0.0	0.0	0.1
61	ASN	0.1	-0.2	-1.0	-0.3
62	SER	0.0	-0.1	-0.4	-0.2
63	GLN	-0.1	-1.0	-0.6	-0.6
64	THR	0.0	0.0	0.0	0.0
65	HIS	-0.5	-0.2	-0.7	-0.5
66	GLN	-0.5	-0.1	-0.3	-0.3
67	ALA	-1.0	-0.2	-0.6	-0.6
68	SER	-0.2	0.0	-0.5	-0.2
69	LEU	-1.3	-0.1	-1.6	-1.0
70	SER	0.1	0.1	-0.2	0.0
71	LYS	-2.1	-1.9	-6.4	-3.5
72	GLN	-0.4	0.5	2.1	0.7

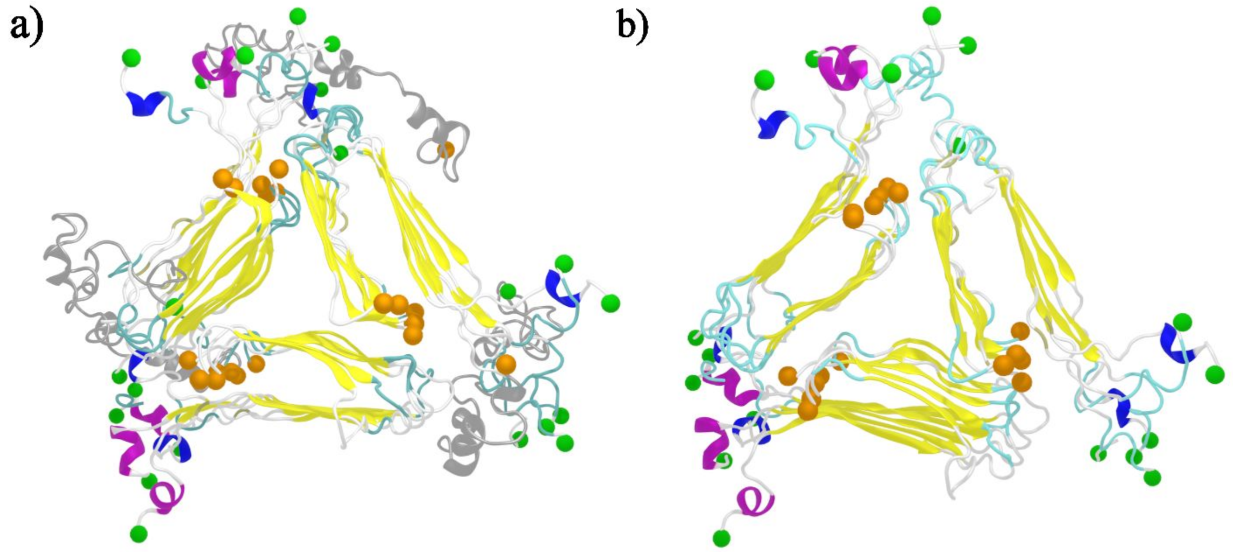

Figure S12: Starting configuration of Aβ40 fibril in the presence (a) and absence (b) of TAT protein. In both systems, unresolved N-terminal regions (resid 1-8) of Aβ fibril are modeled. TAT proteins are colored in grey, and the N- and C-terminal residues are indicated by green and orange spheres, respectively.

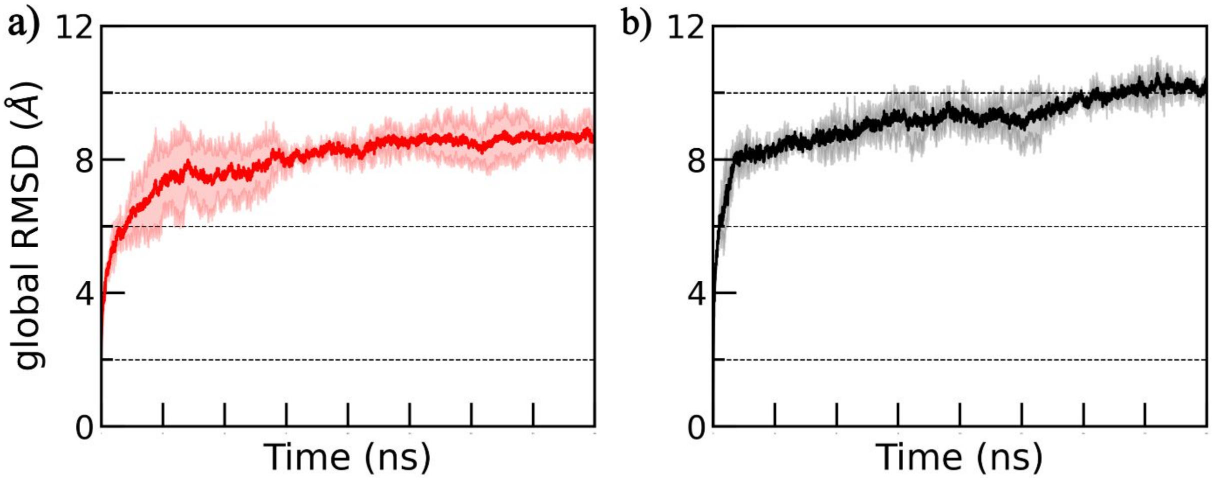

Figure S13: Time evolution of global RMSD (in Å) of Aβ fibril in the presence (a) and absence (b) of TAT protein. In both systems, unresolved N-terminal regions (resid 1-8) of Aβ fibril are modeled. RMSD calculation was performed over the experimentally solved regions (resid 9-40), and are averaged over the two independent trajectories for each system. The shaded region represents the standard deviation.

Table S11: Binding effective energy (ΔG_{eff}) between each TAT protein and Aβ fibril with disordered N-terminus. Data are averaged over the final 50.0 ns of two independent trajectories. Standard deviations are presented within braces. Values are reported in unit of kcal/mol.

Aβ fibril-TAT complex	ΔE_{elec}	ΔE_{vdW}	$\Delta G_{sol\text{-}pol}$	$\Delta G_{sol\text{-}np}$	ΔH_{MM}	ΔG_{solv}	ΔG_{eff}
run 1	-6876.8(363.1)	-128.4(15.4)	6834.7(355.6)	-23.2(2.7)	-7005.3(367.5)	6811.5(354.0)	-193.7(18.6)
run 2	-6217.7(347.0)	-136.2(49.4)	6210.2(313.0)	-23.3(6.7)	-6354.0(309.6)	6186.9(317.5)	-167.0(25.9)
mean	-6547.3(484.5)	-132.3(36.8)	6522.5(458.0)	-23.2(5.1)	-6679.6(470.7)	6499.2(458.9)	-180.4(26.2)
